# DDR1-induced neutrophil extracellular traps drive pancreatic cancer metastasis

**DOI:** 10.1172/jci.insight.146133

**Published:** 2021-09-08

**Authors:** Jenying Deng, Yaan Kang, Chien-Chia Cheng, Xinqun Li, Bingbing Dai, Matthew H. Katz, Taoyan Men, Michael P. Kim, Eugene A. Koay, Huocong Huang, Rolf A. Brekken, Jason B. Fleming

**Affiliations:** 1 Department of Surgical Oncology,; 2 Department of Experimental Therapeutics,; 3 Department of Molecular and Cellular Oncology,; 4 Functional Genomics Core,; 5 Genetics, Division of Basic Science Research,; 6 Department of Radiation Oncology,; 7 Department of Surgery and Hamon Center for Therapeutic Oncology Research, Division of Surgical Oncology, and; 8 Department of Pharmacology, The University of Texas Southwestern Medical Center, Dallas, Texas, USA.; 9 Department of Gastrointestinal Oncology, H. Lee Moffitt Cancer Center, Tampa, Florida, USA.

**Keywords:** Oncology, Cytokines, Neutrophils, Signal transduction

## Abstract

Pancreatic ductal adenocarcinoma (PDAC) tumors are characterized by a desmoplastic reaction resulting in dense deposition of collagen that is known to promote cancer progression. A central mediator of protumorigenic collagen signaling is the receptor tyrosine kinase discoid domain receptor 1 (DDR1). DDR1 is a critical driver of a mesenchymal and invasive cancer cell PDAC phenotype. Previous studies have demonstrated that genetic or pharmacologic inhibition of DDR1 reduces PDAC tumorigenesis and metastasis. Here, we investigated whether DDR1 signaling has cancer cell nonautonomous effects that promote PDAC progression and metastasis. We demonstrate that collagen-induced DDR1 activation in cancer cells is a major stimulus for CXCL5 production, resulting in the recruitment of tumor-associated neutrophils (TANs), the formation of neutrophil extracellular traps (NETs), and subsequent cancer cell invasion and metastasis. Moreover, we have identified that collagen-induced CXCL5 production was mediated by a DDR1/PKC**θ**/SYK/NF-**κ**B signaling cascade. Together, these results highlight the critical contribution of the collagen I–DDR1 interaction in the formation of an immune microenvironment that promotes PDAC metastasis.

## Introduction

Pancreatic ductal adenocarcinoma (PDAC) is now the third leading cause of cancer death in the United States. The majority of patients with PDAC are found to have metastatic disease at diagnosis, and only approximately 10% of patients survive over 5 years (1, 2). A major contributor to the dismal prognosis of PDAC is its unique stroma. PDAC is characterized by a desmoplastic reaction accompanying the progression of the disease, resulting in the deposition of a dense extracellular matrix (ECM) (3). One of the major ECM components is collagen, which can promote cancer cell survival and facilitate invasion (4, 5). However, the mechanisms through which collagen functions to promote PDAC progression are not clear. The discoid domain receptor (DDR) family, which includes DDR1 and DDR2, is the only receptor tyrosine kinase family that is specifically activated by fibrillar collagens (5, 6). Upon ligation with collagen, DDRs undergo autophosphorylation and propagate downstream signaling. DDRs have been shown to regulate cancer cell survival, adhesion, proliferation, motility, and invasion in different settings (7). In PDAC, elevated expression of DDR1 is negatively associated with clinical outcomes (8). In addition, collagen-DDR1 signaling can induce an invasive phenotype in pancreatic cancer cells through an epithelial-mesenchymal transition (EMT) (9, 10). Therefore, DDR1 might be a critical mediator of collagen-driven tumorigenesis in PDAC. This is supported by a recent study involving genetically ablated DDR1 in a genetically engineered mouse model (GEMM) of PDAC (11). As a result, the progression of tumors was significantly delayed, and the tumors failed to progress into an undifferentiated phenotype. Moreover, the metastases of the DDR1-deficient tumors were also significantly reduced. We reported in an earlier study that the pharmacological inhibition of DDR1 activation by a novel small-molecule inhibitor, 7rh benzamide, inhibited tumorigenesis and enhanced chemosensitivity in orthotopic xenografts and autochthonous pancreatic tumors (12).

Although DDR1 induces an invasive cancer cell phenotype that contributes to invasion, metastasis, and therapy resistance, whether DDR1 can mediate a communication between cancer cells and stromal cells and alter the tumor microenvironment (TME) is poorly understood. This is particularly important because metastasis is a complex, multistep process requiring cancer cell migration and survival in a distant organ (13). Therefore, a TME that facilitates the distant travel and seeding of cancer cells is essential for tumor progression. The recruitment of immunosuppressive stromal cells, including tumor-associated macrophages (TAMs) and tumor-associated neutrophils (TANs), by cancer cells is a major contributor to a metastasis-permissive TME (14). As a critical component of the innate immune system, neutrophils are first responders to infection and injury (15). Through the generation of reactive oxidants and the activation of granular constituents and neutrophil extracellular traps (NETs), neutrophils target microbes and prevent their dissemination (16). In cancer, there is growing evidence that TANs can enhance tumor progression through NETs (17). NETs are long, thin-stranded, web-like extracellular fibers formed by neutrophils, consisting of chromatin DNA filaments and specific proteins, such as lactoferrin, myeloperoxidase (MPO), histones, and neutrophil elastase. Recent studies have shown that NETs are highly associated with metastasis in different cancer types (18–23). NETs can remodel the stroma to help tumor cells invade and induce thrombosis formation, which in turn help tumor cell clusters progress and metastasize (21, 24, 25). NETs also capture and induce apoptosis of cytotoxic T cells (26). Moreover, a recent study has shown that NETs can activate CCDC25 on cancer cells and enhance cell motility (22). Despite the important functions of TANs and NETs during cancer metastasis, the pathways that cancer cells use to induce NET formation remain unclear.

In this study, we investigated whether cancer cells exploit collagen-DDR1 signaling to communicate with other stromal cells and modulate the TME to promote PDAC progression and metastasis. The results demonstrate that the activation of cancer cell DDR1 by collagen is an essential step for TAN infiltration and NET formation. Specifically, we identified CXCL5 as a key chemokine in collagen I-induced NET formation and show that the pharmacologic blockade of DDR1 effectively prevents collagen induction of CXCL5, subsequent NET formation, and cancer cell invasion. Moreover, we also identified that collagen-induced CXCL5 production is mediated by a PKCθ/SYK/NF-κB signaling cascade downstream of activated DDR1. Taken together, we conclude that collagen stimulates pancreatic cancer cells to produce CXCL5 through a DDR1/PKCθ/SYK/NF-κB pathway, and as a result, CXCL5 induces TANs to form NETs and promote cancer cell invasion and metastasis.

## Results

### DDR1 drives metastasis in PDAC.

**Figure 1:**
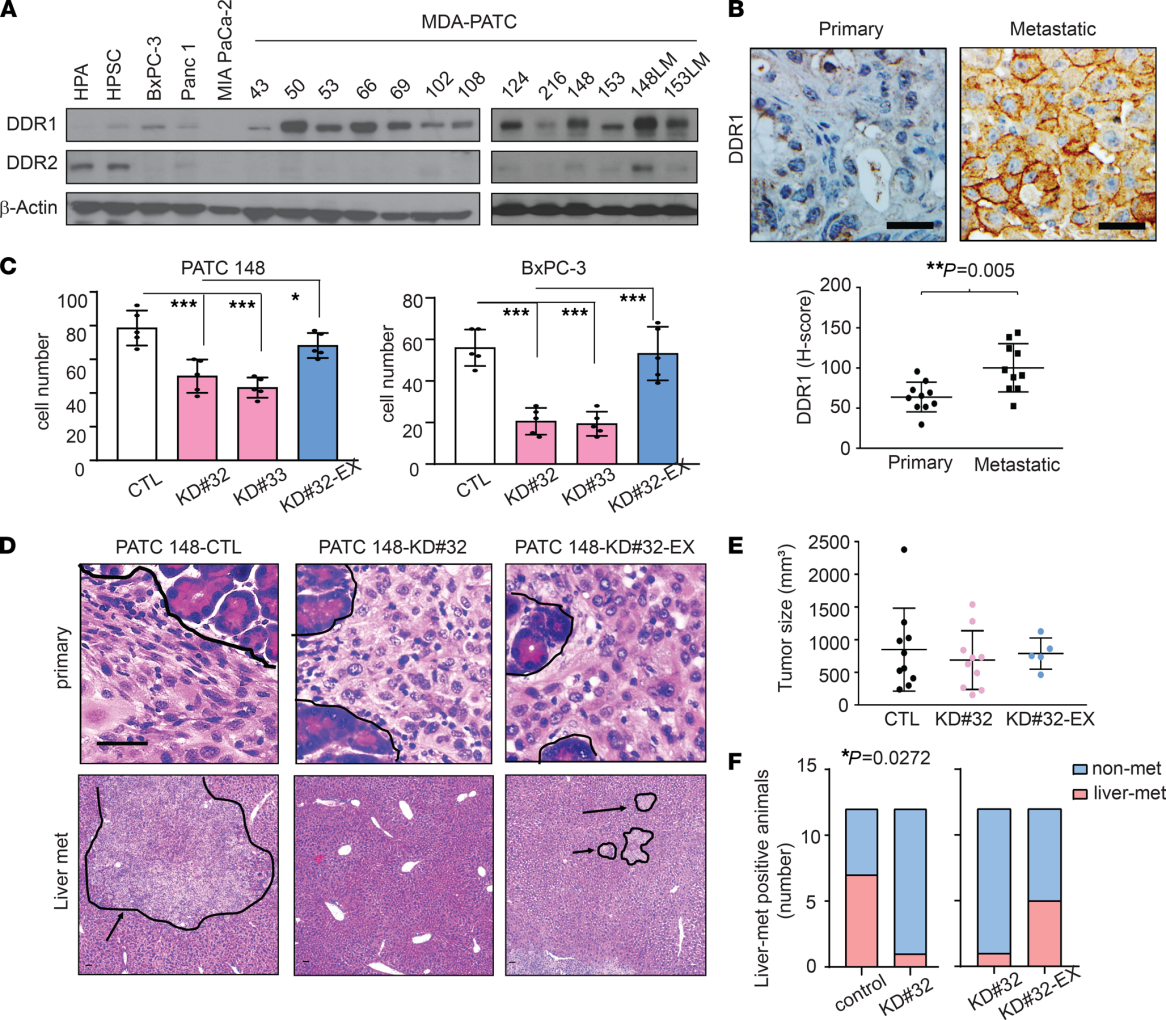
DDR1 induces liver metastasis in pancreatic cancer. (**A**) DDR1 and DDR2 expression were analyzed by western blotting in 2 fibroblasts, 14 primary PDAC cell lines, and 2 metastatic PDAC cell lines, in 3 independent experiments. (**B**) DDR1 was observed at PDX tumors derived from metastatic or primary human PDAC tumors by IHC staining using anti–human DDR1 antibody and identified using PE Vectra3. Scale bar: 50 μm. The H-score of DDR1 quantification was displayed as DBA signals by inForm software. *n* = 10, unpaired 2-tailed Student’s *t* test. **P < 0.01. (**C**) Cell invasion assay in MDA-PATC 148 cells with knockdown or reexpression DDR1 were used by Matrigel transwell chamber. The invading cells in each chamber were counted under a fluorescence microscope after cultured 18 hours, and the average number of cells was calculated based on the number of cells found in 6 fields per chamber. Data are mean ± SD. *n* = 5, 3 independent experiments; 1-way ANOVA with Sidak post hoc testing. *P < 0.05; ***P < 0.001. (**D**–**F**) Mice were orthotopically injected with MDA-PATC 148 (control, DDR1–deficient or DDR1-reexpression clones) cells for 9 weeks. (**D**) H&E staining of pancreas and liver section. *Arrow*: region of tumor. *n* = 12. Scale bar: 50 μm. (**E**) Tumor size measurement in pancreas. Unpaired 2-tailed Student’s *t* test. (**F**) The numbers of liver-met. *n* = 12; Fisher’s exact test. *P < 0.05.****

Recent evidence demonstrates that the genetic ablation of DDR1 in a GEMM of PDAC resulted in a significant reduction of metastasis (11). To further validate this result, we exploited widely used human PDAC cell lines, primary human PDAC cell lines (MDA-PATC) from patient-derived xenograft (PDX) tumors generated in our laboratory (27). We first examined the expression of DDRs in PDAC cell lines and confirmed that 14 of the cell lines expressed high levels of DDR1, while only several expressed DDR2 (Figure 1A). We orthotopically implanted 2 cell lines with robust DDR1 expression, MDA-PATC 148 and MDA-PATC 153, into nude mice and harvested liver metastases, which were used to generate matched metastatic cell lines (PATC 148LM and 153LM). We compared MDA-PATC 148LM and 153LM cells with the matched parental lines and found that the metastatic clones expressed higher levels of DDR1 (Figure 1A). Comparison of PDX tumors derived from metastatic or primary human PDAC tumors also revealed that PDX derived from metastatic tumors expressed higher levels of DDR1 than PDX derived from primary tumors (Figure 1B).

To confirm DDR1-induced cancer cell invasion and metastasis, we generated stable DDR1-deficient clones using different shRNAs against DDR1 in MDA-PATC 148 (MDA-PATC 148^KD#32^ and MDA-PATC 148^KD#33^) and BxPC-3 (BxPC-3^KD#32^ and BxPC-3^KD#33^) cells (Supplemental Figure 1A; supplemental material available online with this article; https://doi.org/10.1172/jci.insight.146133DS1). In addition, we rescued DDR1 expression by introducing an shRNA-resistant DDR1 construct in MDA-PATC 148^KD#32^ (MDA-PATC 148^KD#32-exDDR1^) and BxPC-3^KD#32^ (BxPC-3^#32-exDDR1^) cells (Supplemental Figure 1A). The loss of DDR1 resulted in a reduction of invading cells in each cell line and an effect that was rescued by DDR1 reexpression (Figure 1C). Upon orthotopic implantation of MDA-PATC 148^CTL^, MDA-PATC 148^KD#32^, and MDA-PATC 148^KD#32-exDDR1^ cells, we found that DDR1 knockdown in the cancer cells had no effect on primary tumor growth but resulted in a significant reduction in the incidence of liver metastasis (WT, 58.33%; KD^#32^, 12.5%; *P* value = 0.0272 in Fisher’s exact test) (Figure 1, D–F).

### DDR1 induces CXCL5 production and Ly6G^+^ neutrophil infiltration.

**Figure 2:**
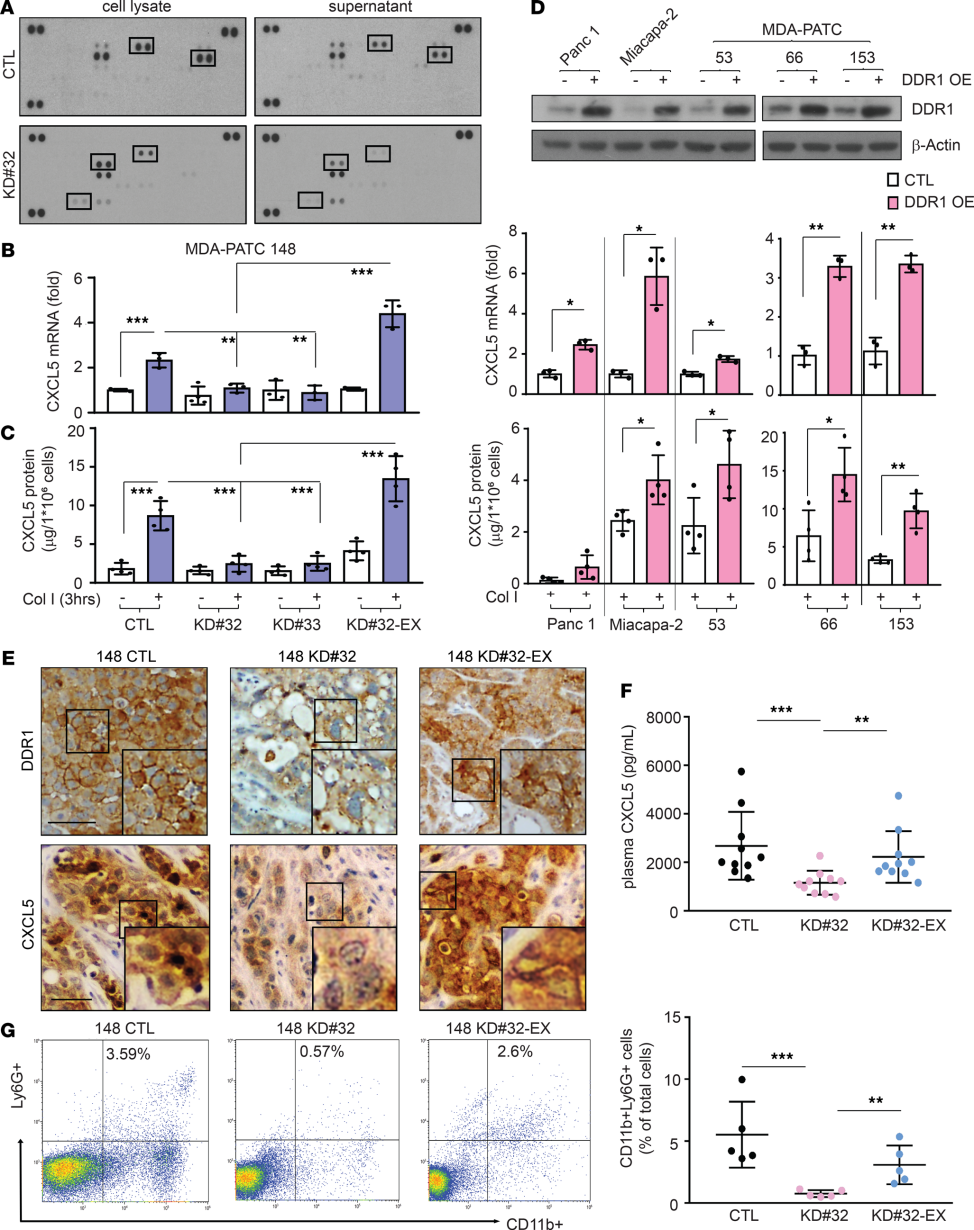
DDR1 induces CXCL5 production in pancreatic cancer cells. (**A**) Chemokine array analysis in cell lysate and supernatant of MDA-PATC 148 cells with knockdown DDR1. (**B **and** C**) MDA-PATC 148 cells with knockdown or reexpressed DDR1 were treated with collagen I for 3 hours. (**B**) CXCL5 mRNA level by using real-time PCR. (**C**) CXCL5 protein level by using ELISA. (**D**) CXCL5 expression in overexpressed DDR1 in 5 pancreatic cancer cell lines. Upper:** DDR1 levels were checked by western; middle:** CXCL5 mRNA level were detected by real-time PCR;** lower: CXCL5 protein levels were analyzed by ELISA. (**E**–**G**) Mice were orthotopically injected with MDA-PATC 148 (control, DDR1-deficient or DDR1-reexpression clones) cells for 9 weeks. (**E**) IHC staining with anti-DDR1 (upper panel) and anti-CXCL5 (bottom panel) antibodies in pancreas. (**F**) ELISA showed CXCL5 level in plasma harvest from mice. (**G**) FACS by using anti-CD11b and anti-Ly6G antibodies to determine the presence of CD11b^+^Ly6G^+^ neutrophils infiltration in pancreas. (**B**–**D**) Data are mean ± SD. *n* = 3–4, 3 independent experiments; (**B **and** C**) 1-way ANOVA with Sidak post hoc testing; (**D**) Unpaired 2-tailed Student’s *t* test. *P < 0.05; **P < 0.01. (**F **and** G**) *n* = 5–10 mice, data performed in triplicate; 1-way ANOVA with Sidak post hoc testing. **P < 0.01; ***P < 0.001. Data show signal after membrane exposed to x ray film for 2 minutes.

To investigate the influence of cancer cell DDR1 signaling on the TME, we screened for cancer cell DDR1-induced cytokine production using a human chemokine antibody array. We found that the activation of DDR1 by collagen induced the production of 4 candidate factors, CD130, CXCL8, CXCL5, and MCP-1, which were reduced by knocking down DDR1 (Figure 2A). We then validated that CXCL5 was a DDR1-induced factor by quantitative PCR (qPCR) assays (Supplemental Figure 1B). The exposure of parental cancer cells to collagen I for 3 hours increased mRNA and protein levels of CXCL5 in MDA-PATC 148 and BxPC-3 cells, which was diminished in knockdown DDR1 cells (Figure 2, B and C, and Supplemental Figure 1C). In addition, the mRNA and protein expression of CXCL5 was rescued after reexpression of DDR1 in MDA-PATC 148^KD#32^ and BxPC-3^KD#32^ cells (Figure 2B and Supplemental Figure 1C). To confirm this was a DDR1-mediated effect, we overexpressed DDR1 in an additional 5 human pancreatic cancer cell lines, which resulted in a significant increase of CXCL5 expression upon collagen activation (Figure 2D).

**Figure 3:**
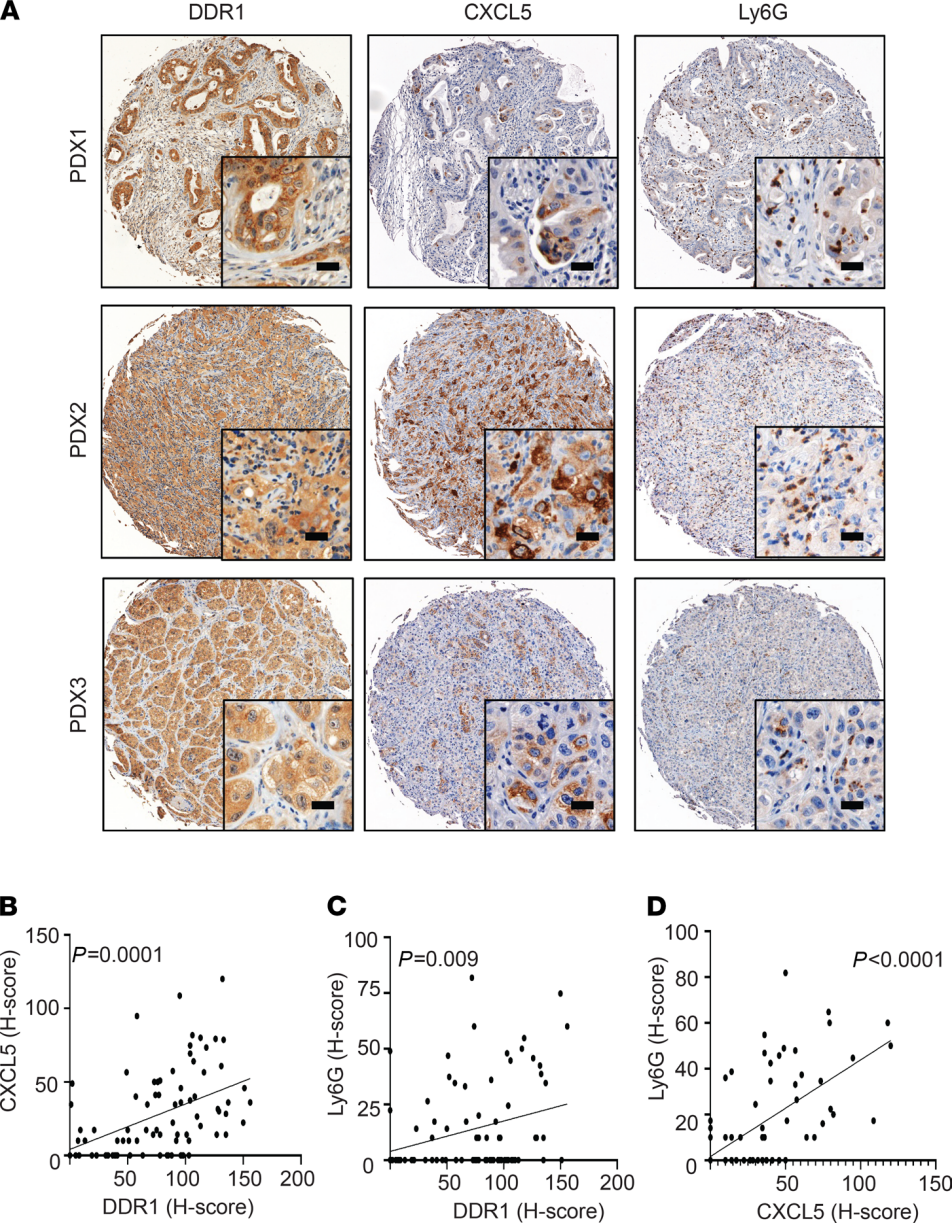
The correlation of DDR1, CXCL5, and neutrophils infiltration at tissue microarray (TMA) in PDX tumors. (**A**) IHC staining showed DDR1, CXCL5, and Ly6G^+^ neutrophils infiltration at PDX tumors and identified using PE Vectra3. Scale bar: 50 μm. (**B**–**D**) Pearson’s correlation showed relationship of DDR1, CXCL5, and Ly6G by using H-score, which quantified the DBA signals by inForm software. *n* = 82.

CXCL5, also known as epithelial-derived neutrophil-activating peptide 78 (ENA-78), has been previously shown to induce TAN infiltration and increase metastatic risk in hepatocellular carcinoma (28). We sought to investigate whether DDR1-derived CXCL5 is associated with TAN infiltration in PDAC. We first measured the level of CXCL5 and Ly6G^+^ neutrophil infiltration in tumors derived from MDA-PATC 148 variants (MDA-PATC 148^CTL^, MDA-PATC 148^KD#32^, and MDA-PATC 148^KD#32-exDDR1^). Knocking down DDR1 reduced the level of CXCL5 in the primary tumor and plasma (Figure 2, E and F). Importantly, we also observed a reduction of CD11b^+^Ly6G^+^ neutrophil infiltration in DDR1-knockdown tumors (Figure 2G). Reexpression of DDR1 (MDA-PATC 148^KD#32^-ex-DDR1) rescued CXCL5 levels in the tumor and plasma as well as CD11b^+^Ly6G^+^ TAN infiltration (Figure 2, E–G). To verify this association in a clinically relevant setting, we performed IHC on a tissue microarray of pancreatic cancer PDXs. Scoring from 82 tumor samples identified a positive correlation between DDR1 expression and CXCL5 production (Pearson’s *r* = 0.4460; 95% CI, 0.2535–0.6045) and Ly6G^+^ TAN infiltration (*r* = 0.2840; 95% CI, 0.07144–0.4720) (Figure 3, A–C). As expected, a positive correlation was also observed between CXCL5 and the infiltration of Ly6G^+^ TANs (*r* = 0.6403; 95% CI, 0.4916–0.7527) (Figure 3, A and D). To confirm the results were not an artifact of immunodeficient mice necessary for PDX generation, we repeated the experiments in a syngeneic model. KP^wm^C cells (congenic mouse PDAC cells derived from *Kras^LSL.G12D/+^*; *p53^R172H/+^*; *and Pdx1^CreTg/+^* [*KP^wm^C*] mice) with stable DDR1 knockdown were generated (KP^wm^C^KD#588^ and KP^wm^C ^KD#809^) (Supplemental Figure 2A) and orthotopically implanted into immunocompetent C57BL/6J mice. Similar to the xenograft models, DDR1-deficient KP^wm^C orthotopic tumors consistently demonstrated decreased CD45^+^CD11b^+^Ly6G^+^ neutrophil infiltration and fewer liver metastases (WT, 80%; KD^#588^, 0%; and KD^#809^, 20%) as well as the level of CXCL5 in plasma (Supplemental Figure 2, B–D). Together, these results suggest that DDR1 on cancer cells drives CXCL5 production, CD45^+^CD11b^+^Ly6G^+^ TAN infiltration, and liver metastasis.

### DDR1-induced NET formation enhances pancreatic cancer cell invasion.

**Figure 4:**
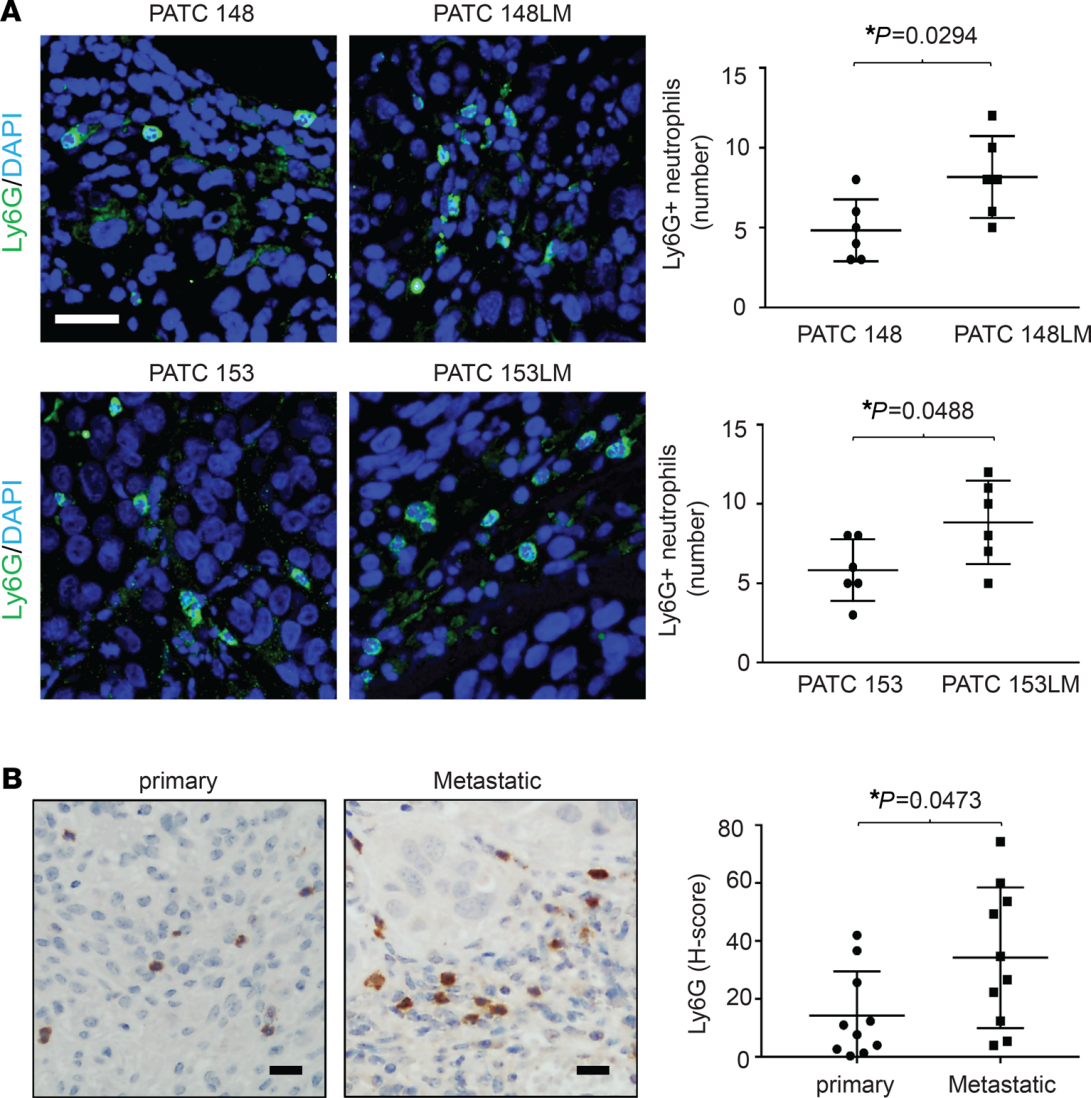
Metastatic tumors recruit more Ly6G^+^ neutrophils infiltration than primary tumors. (**A**) Ly6G^+^ neutrophils were observed at tumors derived from primary and match liver-met cell lines by immunofluorescence staining using anti-Ly6G (green) and DAPI (blue) with a fluorescence microscope. Scale bar: 50 μm. The number of neutrophils were counted in x20 field, 6 fields per slice. Data are mean ± SD. *n* = 5 mice, unpaired 2-tailed Student’s *t* test. *P < 0.05. (**B**) Ly6G^+^ neutrophils were observed at PDX tumors derived from metastatic or primary human PDAC tumors by IHC staining using anti-Ly6G antibody and identified using PE Vectra3. Scale bar: 50 μm. The H-score of Ly6G quantification was displayed as DBA signals in x20 field, 6 fields per slice by inForm software. Data are mean ± SD. *n* = 10, unpaired 2-tailed Student’s *t* test. *P < 0.05.

We then asked if DDR1-induced TAN infiltration directly enhanced the metastatic capability of cancer cells. We first observed a higher level Ly6G^+^ TANs within the TME of tumors derived from cell lines selected for liver metastasis, PATC 148LM and 153LM, compared with tumors derived from the parental lines (Figure 4A). In addition, PDXs derived from metastatic PDAC tumors were found to have higher numbers of Ly6G^+^ TANs than PDXs derived from primary tumors (Figure 4B).

**Figure 5:**
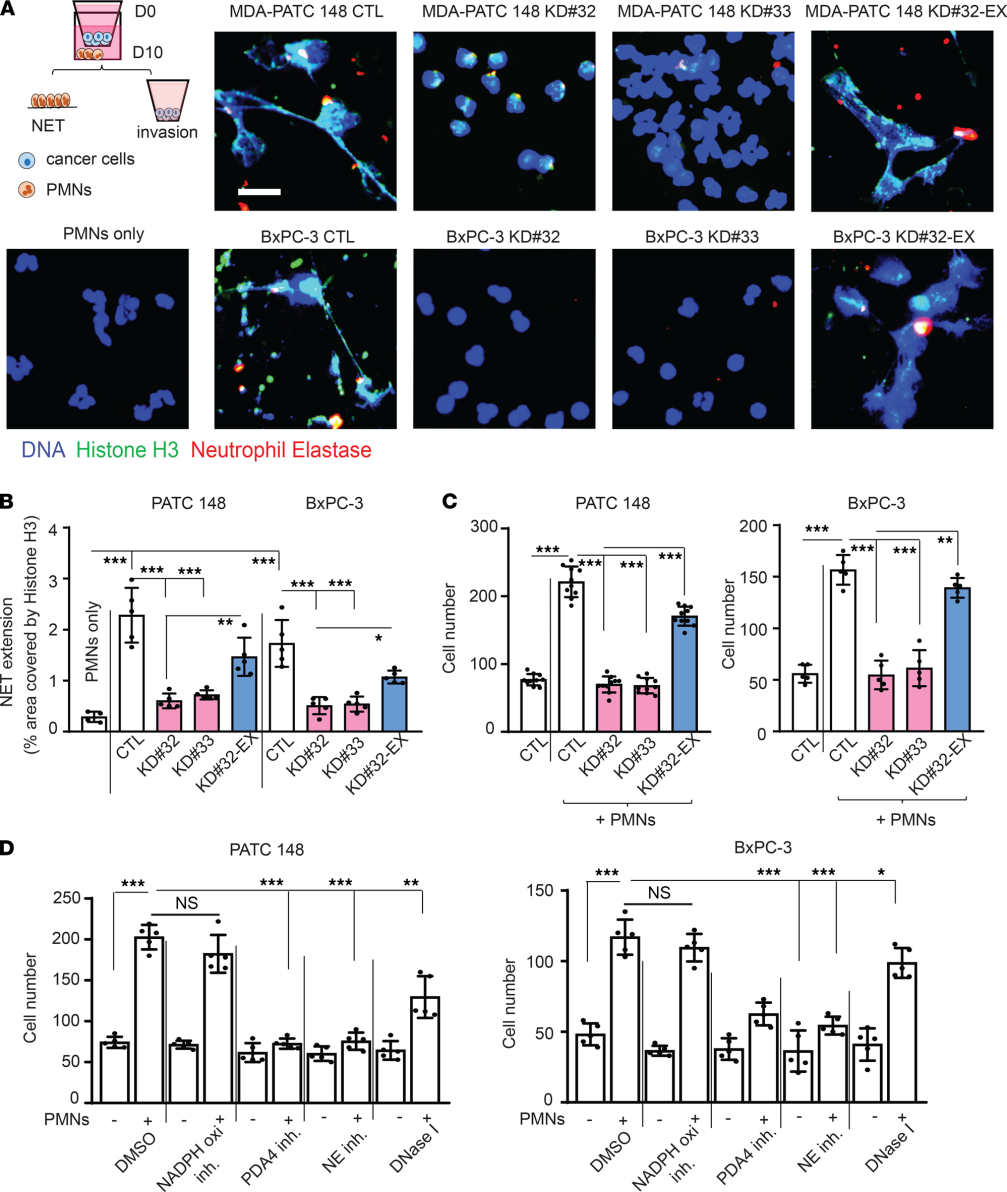
DDR1-positive pancreatic cancer cells mediated NET formation from neutrophils and enhanced cancer cell invasion. **(A**–**D**) Human neutrophils were cocultured with DDR1 knockdown or reexpression of MDA-PATC 148 or BxPC-3 by Matrigel transwell chamber, with or without NADPH oxidase inhibitor, PDA4 inhibitor, NE inhibitor, and Dase I treatment for 18 hours. (**A**) NET structures were analyzed by immunofluorescence staining using DAPI (blue), anti-NE (red), and anti-histone H3 (green) mAbs. Scale bar*:* 50 μm. (**B**) The NET quantification is displayed as NET histone area (μm^2^) per x40 field, 6 fields per group. (**C **and** D**) The number of invaded cells analyzed by immunofluorescence staining using DAPI and calculated based on the number of cells found in 6 fields per chamber. All the data are mean ± SD. *n* = 5, 3 independent experiments; 1-way ANOVA with Sidak post hoc testing. *P < 0.05; **P < 0.01; ***P < 0.001.

Recent studies have demonstrated NET formation is a major driver of metastasis in breast cancer (21). In PDAC, NETs have also been frequently observed in tumor tissues (18). Thus, we investigated whether NETs contributed to DDR1-induced cancer cell invasion. NET formation requires the generation of reactive oxygen species and nicotinamide adenine dinucleotide phosphate (NADPH) oxidase activity as well as the activation of peptidylarginine 4 (PAD4) that promotes the decondensation of nuclear DNA by histone citrullination and the release of MPO/neutrophil elastase (NE) from azurphilic granules (29, 30). These events can be triggered in vitro by exposing neutrophils to phorbol myristate acetate (PMA) (31). We cocultured neutrophils harvested from the blood of patients with untreated PDAC, with MDA-PATC 148 or BxPC-3 cells for 18 hours using a Matrigel-coated transwell chamber and found increased NET formation and citrullinated histone H3 expression (Figure 5, A and B, and Supplemental Figure 3A). Histone H3 citrullination and NET formation were significantly reduced when the experiments were performed using PDAC cells lacking DDR1 (Figure 5, A and B, and Supplemental Figure 3A) and were rescued when DDR1 expression was restored (Figure 5, A and B). When compared with cancer cells alone, the addition of neutrophils resulted in an almost 3-fold increase in invasion of MDA-PATC 148 and BxPC-3 cells (Figure 5C). Knocking down DDR1 in the cancer cells significantly reduced neutrophil-mediated cancer cell invasion (Figure 5C). In contrast, the reexpression of DDR1 recovered the effect of neutrophil-mediated cancer cell invasion in MDA-PATC 148^KD#32-exDDR1^ and BxPC-3^KD#32-exDDR1^ cells (Figure 5C). Interestingly, the inhibition of PDA4 and NE inhibited NET formation and cancer cell invasion in cocultured neutrophils with MDA-PATC 148 and BxPC-3 for 18 hours (Supplemental Figure 3, B and C, and Figure 5D). However, NADPH oxidase inhibition had no effect on NETs or cancer cell invasion, and DNase I treatment showed only a partial effect compared with the control (Supplemental Figure 3, B and C, and Figure 5D). Taken together, these data suggest that DDR1 on pancreatic cancer cells induces the formation of NETs, which promote cancer cell invasion through an NAPDH oxidase-independent pathway.

### Cancer cell DDR1-induced CXCL5 mediates NET formation and NET-induced cancer cell invasion.

**Figure 6:**
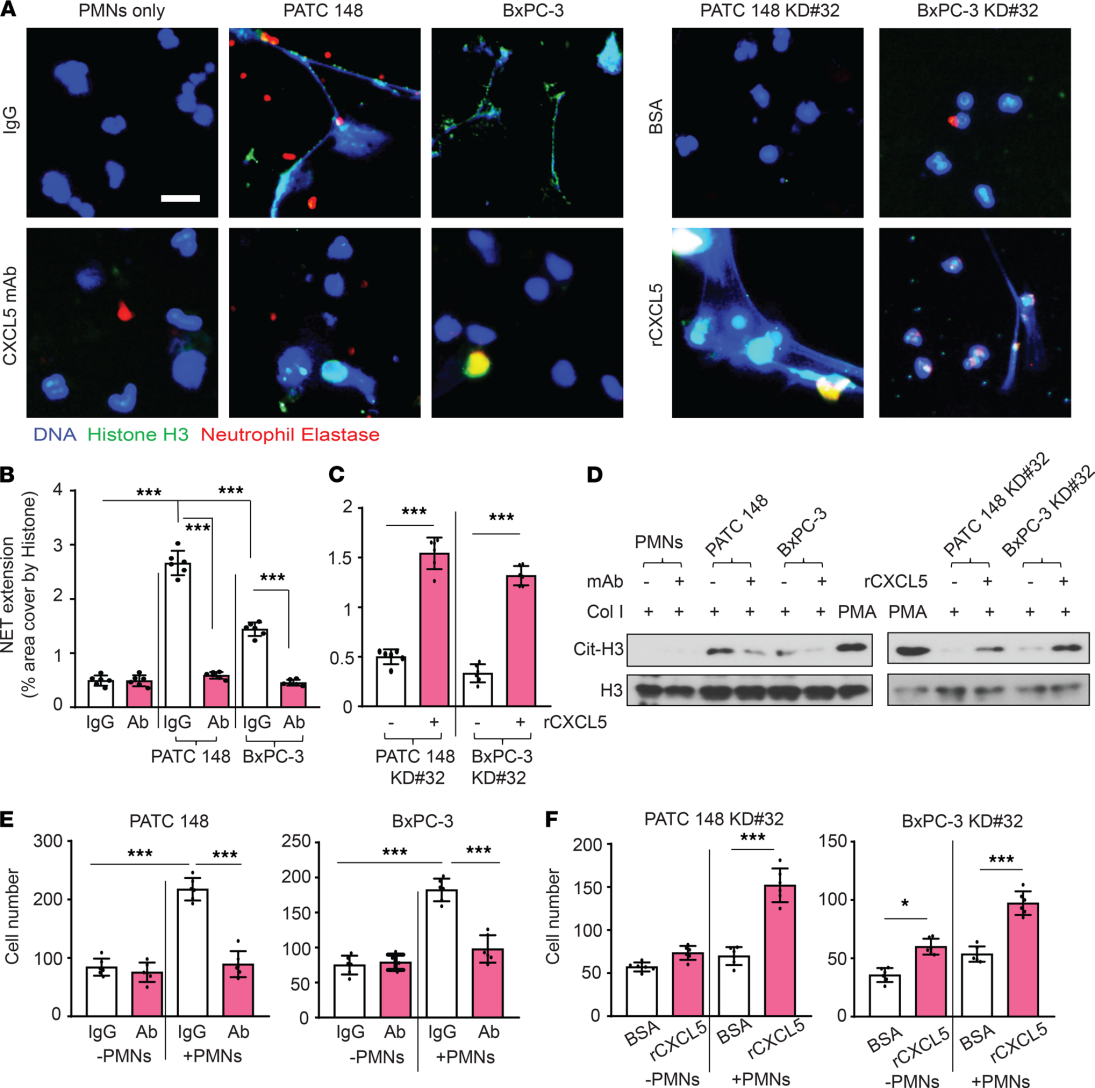
CXCL5 involved in DDR1-mediated NET formation and cancer cell invasion. (**A**–**F**) Human neutrophils were cocultured with DDR1 knockdown or reexpression of MDA-PATC 148 or BxPC-3 by Matrigel transwell chamber, with or without anti-CXCL5 neutralized antibody or recombinant CXCL5 treatment, for 18 hours. (**A**) NET structures were analyzed by immunofluorescence staining using DAPI (blue), anti-NE (red), and anti–histone H3 (green) mAbs. Scale bar: 50 μm. (**B **and** C**) The NET quantification is displayed as NET histone area (μm^2^) per field, 6 fields per group. (**D**) Cit-histone H3 expression were analyzed by western blotting. (**E **and** F**) The number of invaded cells were analyzed by immunofluorescence staining using DAPI and calculated based on the number of cells found in 6 fields per chamber. All the data are mean ± SD. *n* = 5, 3 independent experiments. (**B **and **E**) P values were analyzed by 1-way ANOVA with Sidak post hoc testing. ***P < 0.001. (**C **and** F**) P values were analyzed by unpaired 2-tailed Student’s *t* test. *P < 0.05; ***P < 0.001.

**Figure 7:**
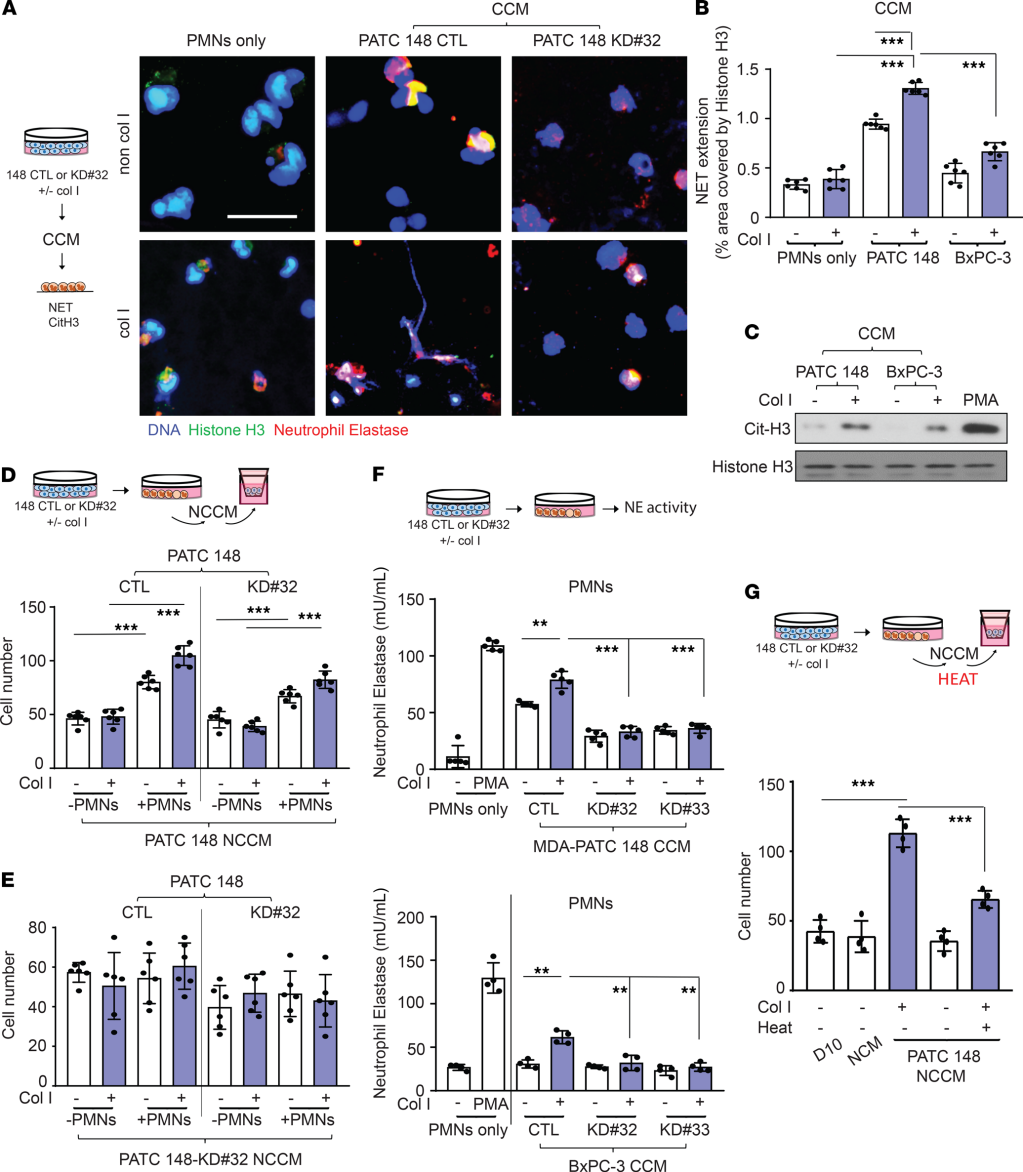
DDR1-positive pancreatic cancer cells mediated NET formation from neutrophils through a soluble factor secretion and enhanced cancer cell invasion. (**A**–**G**) Human neutrophils were cultured with CCM from cancer cells for 18 hours. (**A**) NET structures were analyzed by immunofluorescence staining using DAPI (blue), anti-NE (red), and anti–histone H3 (green) mAbs. Scale bar: 50 μm. (**B**) The NET quantification is displayed as NET histone area (μm^2^) per field, 6 fields per group. (**C**) Cit-histone H3 expression were analyzed by western blotting. (**D **and** E**) The number of invaded cells analyzed by immunofluorescence staining using DAPI and calculated based on the number of cells found in 6 fields per chamber. (**F**) Neutrophils Elastase activity were showed in human neutrophils with CCM treatment for 18 hours. (**G**) The number of invaded cells analyzed by immunofluorescence staining using DAPI and calculated based on the number of cells found in 6 fields per chamber. All the data are mean ± SD. *n* = 4–5, 3 independent experiments; 1-way ANOVA with Sidak post hoc testing. **P < 0.01; ***P < 0.001.

To examine the function of CXCL5 in DDR1-mediated NET formation and cancer cell invasion, the invasion assay was repeated in the presence of a CXCL5-neutralizing monoclonal antibody or recombinant CXCL5. The CXCL5-neutralizing antibody significantly reduced MDA-PATC 148 and BxPC-3 cell–induced histone H3 citrullination, NET formation, and cancer cell invasion (Figure 6, A, B, D, and E). Conversely, recombinant human CXCL5 induced histone H3 citrullination, NET formation, and cancer cell invasion in MDA-PATC 148^KD^ ^#32^ cells (Figure 6, A, C, D, and F). The inhibition of PDA4 and NE blocked recombinant CXCL5-mediated, NET-induced MDA-PATC 148^KD#32^ cell invasion, but NADPH inhibition had no effect (Supplemental Figure 4). To evaluate whether DDR1-induced NET formation and NET-induced cell invasion were mediated by the secretion of soluble factors, we collected conditioned media from 3 in vitro cell culture conditions: cancer cells alone (CCM), neutrophils alone (NCM), or neutrophils exposed to cancer cell-conditioned media (NCCM). NET formation and citrullinated histone H3 was induced by CCM from DDR1-expressing cancer cells exposed to collagen I but not from CCM harvested from DDR1-knockdown cancer cells after collagen I stimulation (Figure 7, A–C). We then repeated the cancer cell invasion assay experiment using NCCM in the presence or absence of collagen I. NCCM from MDA-PATC 148^CTL^/collagen I/neutrophil cultures induced cancer cell invasion in a DDR1-independent manner (Figure 7D). However, NCCM harvested from MDA-PATC 148^KD#32^/collagen I/neutrophil cultures failed to increase cancer cell invasion again, regardless of the DDR1 status (Figure 7E). These carefully designed studies demonstrate that CXCL5 is a soluble factor released into the media from DDR1-positive cancer cells in the presence of collagen; when exposed to CXCL5, neutrophils generate NETs that promote cancer cell invasion. The dependence upon DDR1 is demonstrated by the fact that CCM from DDR1-expressing cancer cell/collagen I cultures stimulates NE activity in neutrophils, but NE activity is significantly reduced after exposure to CCM from DDR1-knockdown clones (Figure 7F). In addition, heat treatment of NCCM harvested from DDR1 intact cancer cell/collagen I/neutrophils results in a reduction of cancer cell invasion (Figure 7G), further suggesting that a secreted protein is responsible for the observed effect.

**Figure 8:**
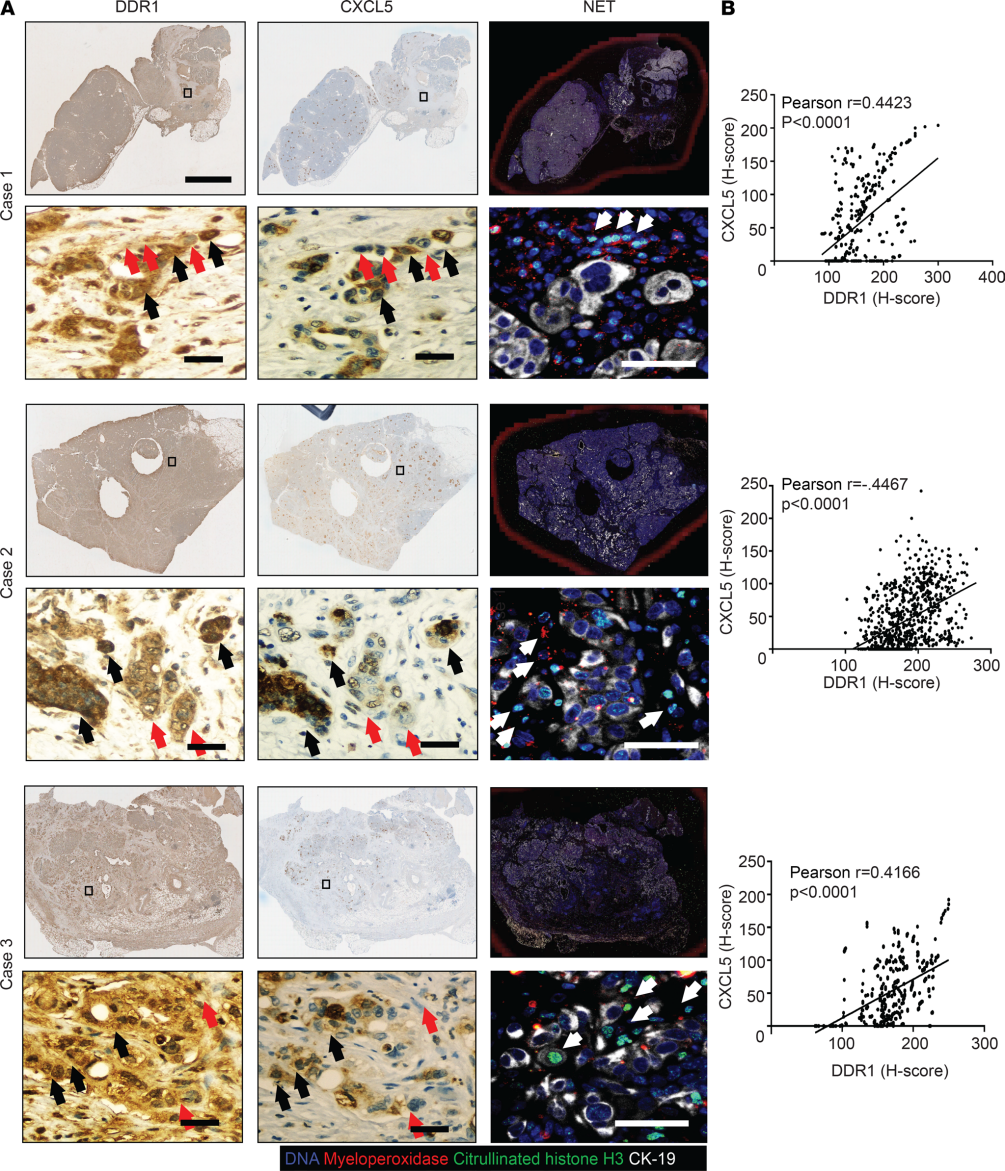
The correlation of DDR1, CXCL5, and NET-like structure in samples of patient with PADC. (**A**) Upper and middle panel: IHC staining showed DDR1, CXCL5 expression in PDAC patient samples, and identified using PE Vectra3. Scale bar: 3 mm and 50 μm. Bottom panel: NET-like structures were analyzed by immunofluorescence staining using DAPI (blue), anti-CK19 (white), anti-MPO (green), and anti–citrullinated histone H3 (red) mAbs in samples of patient with PDAC. Scale bar: 20 μm. (**B**) Pearson’s correlation showed relationship of DDR1 and CXCL5 by using H-score, which quantified the DBA signals by inForm software, *P* < 0.0001.

To examine the DDR1/CXCL5 axis and NET formation in human PDAC, we performed IHC and immunofluorescence staining on primary human PDAC samples. We found that the areas of high DDR1 expression were associated with high intratumoral levels of CXCL5 (black arrows) (Figure 8). Conversely, within the same tumor we observed that regions of low DDR1 expression were associated with decreased CXCL5 expression (red arrow). In addition, we found that the NET-like structures were located around tumor cells (green and red color). Statistically, Pearson’s *r* correlation analysis indicated a positive association between DDR1 and CXCL5 level in 3 cases of pancreatic patient tumor specimens (case 1: *r* = 0.4423; case 2: *r* = 0.4467; and case 3: *r* = 0.4166).

### Cancer cells induce CXCL5 and cause NET formation through the DDR1/PKCθ/NF-κB signaling pathway.

**Figure 9:**
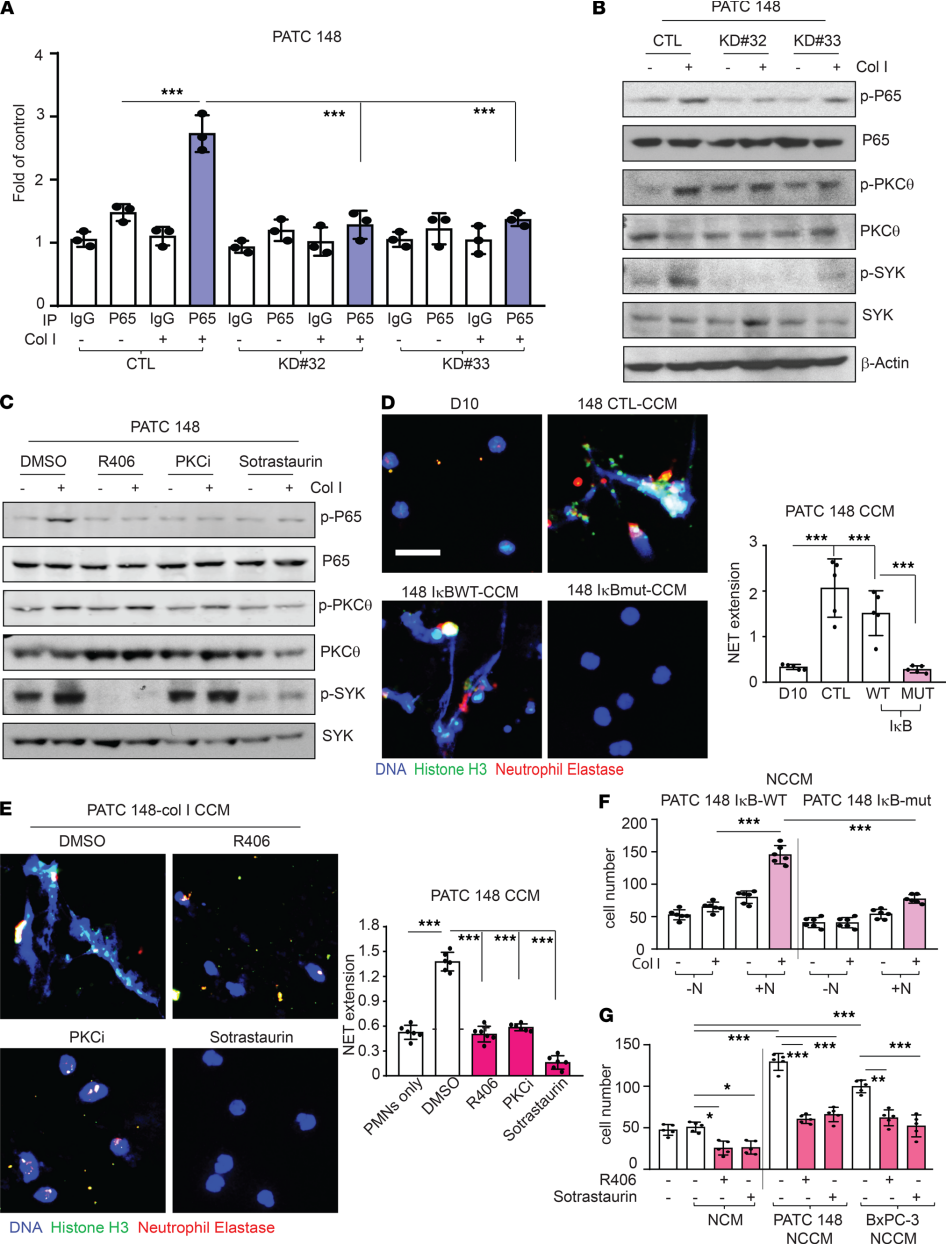
PKCθ/SYK/NF-κB pathway involved in DDR1-induced CXCL5 production, NET formation from neutrophils, and enhanced cancer cell invasion. (**A**) qPCR results were used to quantify enrichment of NF-κB P65 at the CXCL5 promoter using ChIP assay in MDA-PATC 148 cells with DDR1 knockdown. Data are mean ± SD. *n* = 3, 3 independent experiments; 1-way ANOVA with Sidak post hoc testing. ***P < 0.001. (**B**) Phospho-NF-κB P65, phospho-PKCθ, and phospho-SYK were analyzed by western blotting in MDA-PATC 148 cells with DDR1 knockdown. (**C**) Phospho-NF-κB P65, phospho-PKCθ, and phospho-SYK were analyzed by western blotting in MDA-PATC 148 cells with or without SYK inhibitor and PKC inhibitor pretreatment. (**D **and** E**) NET structures were analyzed by immunofluorescence staining using DAPI (blue), anti-NE (red), and anti–histone H3 (green) mAbs. (**D**) In MDA-PATC 148 cells with CCM from MDA-PATC 148 with IκB super-repressor mutation/collagen I, treatment for 18 hours. (**E**) In MDA-PATC 148 cells with MDA-PATC 148, with or without SYK inhibitor and PKC inhibitor pretreatment/collagen I, treatment for 18 hours. Scale bar: 50 μm. The NET quantification is displayed as NET histone area (μm^2^) per field, 6 fields per group. (**F **and** G**) The number of invaded cells were analyzed by immunofluorescence staining using DAPI and calculated based on the number of cells found in 6 fields per chamber. (**F**) In MDA-PATC 148 cells with NCCM from MDA-PATC 148 cells with IκB super-repressor mutation/neutrophils/collagen I, treatment for 18 hours. (**G**) In MDA-PATC 148 cells with NCCM from MDA-PATC 148/collagen I/SYK or PKC inhibitor, treatment for 18 hours. (**D**–**G**) Data are mean ± SD. *n* = 5–6, 3 independent experiments; 1-way ANOVA with Sidak post hoc testing. *P < 0.05;**P < 0.01; ***P < 0.001.

It has been reported that CXCL5 is a downstream product of the NF-κB pathway (32) and that the NF-κB pathway can be activated by DDR1 (33). Therefore, we sought to determine whether the NF-κB pathway is involved in collagen-induced CXCL5 production. ChIP-qPCR assay demonstrated that the exposure of MDA-PATC 148 cells to collagen I induced binding of the NF-κB P65 subunit to the CXCL5 promoter, and this binding was reduced significantly after knockdown of DDR1 (Figure 9A). In addition, the exposure of MDA-PATC 148 and BxPC-3 cells to collagen I for 3 hours induced NF-κB P65 translocation into the nucleus (Supplemental Figure 5A). Consistent with this observation, the activation of NF-κB P65 was prevented by genetic DDR1 knockdown in MDA-PATC 148 and BxPC-3 cells (Supplemental Figure 5A). Furthermore, a phospho-NF-κB pathway array performed after collagen I stimulation of MDA-PATC 148^KD#32^ cells demonstrated that the phosphorylation levels of the top 5 proteins had the greatest decrease (20%–25%) in phosphorylation when compared with parental cells: NF-κB P100/52, SYK, NF-κB P65, ZAP-70, and PKCθ (Supplemental Figure 5B). Recent studies have shown that SYK is highly expressed in multiple cancer cell types and that SYK kinase activity induces cancer cell migration and metastasis (34, 35). Thus, we tested whether DDR1 upregulated CXCL5 through NF-κB, SYK, and PKCθ and found that knockdown of DDR1 decreased collagen I-stimulated NF-κB, SYK, and PKCθ phosphorylation (Figure 9B). Next, we generated an IκB super-repressor mutant clone to block the activation of NF-κB in MDA-PATC 148 (MDA-PATC 148^IκB-MUT^) (Supplemental Figure 5C) and found that this significantly inhibited collagen I-induced CXCL5 production (Supplemental Figure 5D). Additionally, pretreatment with SYK and PKC inhibitors prevented collagen I-induced CXCL5 production and inhibited NF-κB P65 activation in MDA-PATC 148 and BxPC-3 cells (Supplemental Figure 5E and Figure 9C). In addition, PKC inhibitors blocked the effect of collagen I-induced SYK phosphorylation; in contrast, the SYK inhibitor had no effect on collagen I-induced PKCθ activation (Figure 9C). These results suggest that collagen I-induced CXCL5 production is mediated by a DDR1/PKCθ/SYK/NF-κB signaling cascade.

To confirm the contribution of the PKCθ/SYK/NF-κB axis to DDR1-mediated NET formation, we harvested CCM from MDA-PATC 148^IκB-WT^, MDA-PATC 148^IκB-MUT^ cells, and MDA-PATC 148 cells pretreated with SYK or PKC inhibitors after exposure to collagen I for 3 hours and incubated neutrophils with the CCM for 18 hours. CCM from cancer cells incapable of NF-κB activation was associated with decreased NET formation after collagen I stimulation (Figure 9D). In addition, pretreatment with SYK and PKC inhibitors protected against MDA-PATC 148 CCM–induced NET formation (Figure 9E). Finally, we tested whether DDR1-mediated NET-induced cancer cell invasion was through the PKCθ/SYK/NF-κB axis. NCCM harvested from MDA-PATC 148^IκB-WT^/collagen I/neutrophil cultures significantly induced cancer cell invasion while NCCM from MDA-PATC 148^IκB-MUT^/collagen I/neutrophil cultures was associated with diminished cancer cell invasion (Figure 9F). NCCM from MDA-PATC 148 and BxPC-3 cells pretreated with SYK or PKC inhibitors/collagen I/neutrophil cultures also reduced cancer cell invasion (Figure 9G). Together, these results provide evidence that DDR1 stimulates CXCL5 production through a PKCθ/SYK/NF-κB signaling cascade and that CXCL5 from cancer cells induces neutrophils to form NETs and thereby enhances metastasis.

### Targeting DDR1 reduces neutrophil-mediated cancer cell invasion.

**Figure 10:**
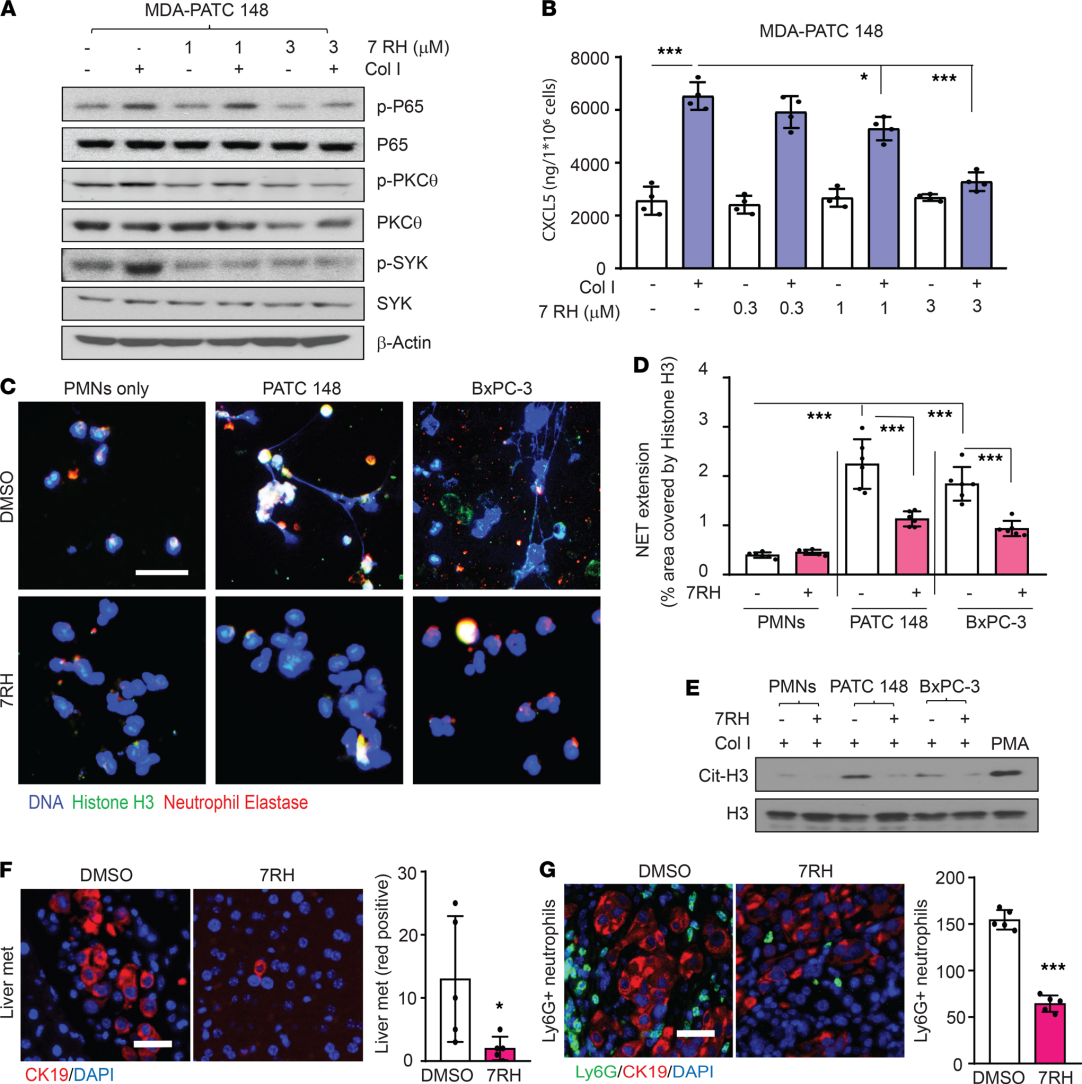
7rh treatment reduced NET formation through inhibition of the DDR1/PKCθ/SYK/CXCL5 axis and reduced cancer metastasis. (**A **and** B**) MDA-PATC 148 cells were pretreated with 7rh for 30 minutes and then with collagen I for 3 hours. (**A**) Phospho-NF-κB P65, phospho-PKCθ, and phospho-SYK were analyzed by western blotting. (**B**) CXCL5 levels were analyzed by ELISA. Data are mean ± SD. *n* = 4, 3 independent experiments; 1-way ANOVA with Sidak post hoc testing. *P < 0.05; ***P < 0.001. (**C**–**E**) Human neutrophils were cocultured with MDA-PATC 148 and BxPC-3 cells by Matrigel transwell chamber for 18 hours. (**C**) NET structures were analyzed by immunofluorescence staining using DAPI (blue), anti-NE (red), and anti–histone H3 (green) mAbs. Scale bar: 50 μm. (**D**) The NET quantification is displayed as NET histone area (μm^2^) per field, 6 fields per group. Data are mean ± SD. *n* = 6, 3 independent experiments; 1-way ANOVA with Sidak post hoc testing. ***P < 0.001. (**E**) Cit-histone H3 expression were analyzed by western blotting. (**F **and** G**) Mice were orthotopically injected with MDA-PATC 148 cells, with or without 3 mg/kg 7rh treatment for 9 weeks. (**F**) Liver metastasis was detected by immunofluorescence staining using DAPI (blue) and anti-CK19 (red) mAbs in liver section. Scale bar: 50 μm. The metastasis quantification is displayed as CK-19 positive signals/per x20 field, 6 fields per group. (**G**) Neutrophils infiltration was detected by immunofluorescence staining using DAPI (blue), anti-CK19 (red), and anti-Ly6G (green) mAbs in pancreas section. Scale bar: 50 μm. The Neutrophils infiltration quantification is displayed as Ly6G positive signals per x20 field, 6 fields per group. Data are mean ± SD. *n* = 5, unpaired 2-tailed Student’s *t* test. ***P < 0.001.

Our previous study demonstrated that the specific DDR1 inhibitor 7rh benzamide can improve the efficacy of standard-of-care chemotherapy in PDAC (12). In the current study, we wanted to determine whether inhibition of DDR1 signaling by 7rh benzamide would result in a reduction of CXLC5 and subsequent NET formation. We found that pretreatment with 3 μM of 7rh benzamide in MDA-PATC 148 and BxPC-3 cells significantly reduced NF-κB, PKCθ, and SYK phosphorylation as well as the production of CXCL5 (Figure 10, A and B, and Supplemental Figure 6A). As predicted, NET formation and cell invasion were significantly decreased in 7rh benzamide–treated cells (Figure 10, C–E, and Supplemental Figure 6B).

To confirm the effect of 7rh benzamide in vivo, we orthotopically implanted MDA-PATC 148 cells into nude mice followed by i.p. treatment with 3 mg/kg/day 7rh benzamide for 9 weeks. 7rh benzamide significantly reduced liver metastasis events (Figure 10F) as well as Ly6G^+^ neutrophil infiltration within the primary tumor (Figure 10G).

## Discussion

Fibrillar collagen is abundant within the microenvironment of primary tumors and has been identified as physically adjacent to cancer cells expressing high levels of DDR1 (36). Here we report a potentially novel mechanism by which a collagen receptor, DDR1, on PDAC cells interacts with type I collagen to attract tumor-associated neutrophils, induce NET formation, and facilitate cancer cell invasion and metastasis. In this study, we mechanistically link DDR1 expression in human PDAC with CXCL5 levels, Ly6G^+^ neutrophil infiltration, NET-like structures, and metastatic events. Importantly, in animal models of PDAC, tumors derived from cancer cells lacking DDR1 had fewer NET-like structures and the animals experienced fewer liver metastases, suggesting that cancer cell-derived DDR1 contributes significantly to NET-induced tumor metastasis.

NETs have been reported to link neutrophils and metastasis (21). Not only neutrophils but also other types of leukocytes, including macrophages, mast cells, and eosinophils, form extracellular traps (a process called ETosis) (37), but NET formation is the most prominent ETosis in cancer. Cancer cell-mediated cytokines and chemokines can prime neutrophils for NET formation by inducing NADPH oxidase activation to support tumor metastasis. NADPH oxidase and PDA4 inhibitors have been reported to significantly decrease tumor cell invasion, suggesting that NET-mediated tumor cell invasion requires neutrophil NADPH oxidase and PDA4 activity (21). Similar to Park and colleagues, we also found that PDA4 and NE activity are important for pancreatic cancer cell–induced NET formation and NET-mediated cancer cell invasion; however, we found that NADPH inhibition had no effect on NET-mediated tumor cell invasion, suggesting that PDAC cells induce NET formation and NET-mediated PATC cell invasion through an NADPH oxidase–independent pathway (Supplemental Figure 3, B and C, and Figure 5D). It has been shown that CXCR2-related chemokines induce NET formation through the NADPH oxidase–independent pathway (38). This is consistent with our findings that CXCL5, a CXCR2 ligand, is expressed by tumor cells in a DDR1-dependent manner and drives neutrophil activation and NET formation (Figure 2; Figure 6, A–D; and Supplemental Figure 1). Recent studies have suggested that NET-associated DNA meshes can catch circulating tumor cells and enhance cell metastasis (19, 39). In addition, NET-associated proteases, NE and matrix metalloprotease 9 (MMP9), awaken dormant cancer cells and facilitate cancer cell metastasis (20, 21). In our studies, we observed a decrease in NE activity through DDR1 knockdown, NE inhibitors, and heat treatment; all of these conditions prevented NET-mediated cancer cell invasion, highlighting the contribution of NE during PDAC cell invasion (Figure 5D; Figure 7, F and G; and Supplemental Figure 4). Taken together, these data support that collagen I–DDR1 interaction induces CXCL5 production by cancer cells, which promotes neutrophil infiltration and NET formation to drive NET-associated cancer cell migration.

In patients, elevated serum CXCL5 levels have been statistically associated with liver metastasis and poor survival (40, 41). CXCL5 recruits neutrophils into the TME (28), and the CXCL5/CXCR2 axis contributes to tumor growth and metastasis through the activation of PI3K/AKT/GSK-3β/Snail signaling to promote EMT (42). However, little is known about the mechanism of CXCL5 production. Our studies demonstrate that the activation of DDR1 signaling by collagen I-induced CXCL5 mRNA and protein expression in PDAC cells in vitro and in vivo. The STAT3 and NF-B pathways are involved in DDR1 downstream signaling and have been reported to induce CXCL5 production (32, 43, 44). However, in our system, the inhibition of STAT3 by cucurbitacin I had no effect on collagen I-induced CXCL5 production (Supplemental Figure 7). For this reason, we focused on the NF-κB pathway and identified a putative signaling cascade from DDR1 to CXCL5 expression via the PKCθ/SYK/NF-κB axis (Figure 9 and Supplemental Figure 6). This is consistent with descriptions of how NF-κB is associated with the hallmarks of cancer, including cancer cell proliferation, protection against apoptosis, and metastasis (45, 46). In addition to NF-κB, PKCθ also promotes cancer metastasis by upregulating EMT and MMP-1 (47, 48), characteristics that are also consistent with our observations.

Constitutive KRAS and NF-κB activation are signature alterations in PDAC (49). Kras^G12D^-induced cytokines and CXCR2-related chemokines have been reported to facilitate myeloid cell infiltration and tumor progression (50–52). In addition, we previously showed that Kras^G12D^*-*activated IL-1α/NF-κB /IL-1α and p62 feedforward loops are necessary for the induction and maintenance of NF-κB activity (53). Here we found that BxPC-3 cells, which are Kras WT, could also produce CXCL5 to drive NET formation. BxPC-3 cells have been reported to bind with high affinity to ECM proteins, including collagen I, and possess invasive capabilities consistent with our observations (54). Nevertheless, it is possible that Kras-mediated NF-κB could drive CXCL5 production, neutrophil infiltration, and NET formation. MDA-PATC 148 cells, which are Kras-mutant, generated higher levels of CXCL5 with associated NET formation and invasion when compared with BxPC-3 cells (Figure 2C; Supplemental Figure 1C; and Supplemental Figure 5, A–C). Although Kras^G12D^ may intrinsically drive NF-κB–CXCL5–mediated NET, our work has identified a potentially novel mechanism by which an abundant stromal molecule, collagen I, can interact with cancer cells through a targetable receptor (DDR1) to induce CXCL5 production, NET formation, and NET-mediated cancer cell invasion, even in the absence of a Kras mutation.

Therapeutic strategies must also account for stromal cells in the TME, which are important in the promotion of metastasis (55). In the current study, we focus on DDR1 and 2 stromal components: collagen I and tumor-associated neutrophils. DDR1 amplification is commonly observed in various cancers (56) and is significantly deregulated in aggressive cancers (57). We observed higher levels of DDR1 in metastatic cell lines compared with matched primary cell lines (Figure 1, A and B). Emerging evidence suggests that DDR1 is crucial for the progression and metastasis of various solid tumors; as such, targeting DDR1 represents a promising therapeutic approach (58–60). This is consistent with our repeated observations of a reduction of cell invasion and metastasis after pharmacologic or genetic inhibition of DDR1 signaling (Figure 1, C–F; Figure 5C; Figure 10, F and G; Supplemental Figure 2, C and D; and Supplemental Figure 6B). The role of neutrophils in solid tumors remains, however, poorly defined. Although TANs support cancer cell progression and invasion, they can also kill cancer cells and bacteria within the TME and are necessary to protect the host from infection (61). For example, the American Society of Clinical Oncology recommends that patients who are undergoing certain chemotherapy regimens receive prophylactic treatment with granulocyte colony-stimulating factor, which stimulates neutrophil production to counteract neutropenia (62). It is possible, however, that inducing neutrophil production may increase the risk of metastatic spread. Recent work suggests that NET production might awaken clinically dormant cancer cells after chemotherapy and drive them to become metastatic (20). Our previous study suggested that a small-molecule inhibitor of DDR1 signaling, 7rh benzamide, would improve the efficacy of standard-of-care chemotherapy in patients with PDAC (12). In this study we found that 7rh significantly reduced cancer cell–induced NET formation in support of our prior observations. Taken together, we suggest that 7rh, when given in therapeutic doses, could increase the sensitivity of cancer cells to conventional chemotherapy and inhibit liver metastasis by blocking NET formation. DDR1 is critical in this process and represents a potential valuable pharmacological target in the treatment of patients with PDAC.

## Methods

### Cell lines.

Pancreatic cancer cell lines — MDA-PATC 43, 50, 53, 66, 69, 102, 108, 124, 148, 153, and 216 — were generated from PDX tumors in our laboratory at various times in the period 2010–2016 (27). The MDA-PATC 148LM and MDA-PATC 153LM cell lines were generated from liver metastases of MDA-PATC 148 and 153 cells *orthotopically implanted* into nude mice, respectively. PANC-1 and BxPC-3 cell lines were obtained from the American Type Culture Collection. WM8865 mouse cells were generated from *Kras^LSL.G12D/+^*; *p53^R172H/+^*; and *Pdx1^CreTg/+^* (*KP^wm^C*) mice in our laboratory. All cells were cultured in DMEM that contained 10% FBS, penicillin (100 units/mL), and streptomycin (100 μg/mL; D10 medium) and had been tested monthly for mycoplasma and found negative.

### Animal studies.

All animal experiments were conducted according to the National Institutes of Health’s Guide for the Care and Use of Laboratory Animals. The study protocol was approved by the University of Texas MD Anderson Cancer Center’s institutional animal care and use committee.

We randomLy assigned 8-week-old nude mice and C57BL/6J mice from the Jackson Laboratory to different groups: nude mice with MDA-PATC 148^CTL^ and MDA-PATC 148^KD#32^; and C57BL/6J mice with KP^wm^C^CTL^, KP^wm^C^KD#588^, and KP^wm^C^KD#809^. We resuspended 1 × 10^5^ cells in 25 μL 1X PBS and added 1 volume of Matrigel (Corning, CB356253). The suspension was then directly injected into the pancreas of mice. Nine weeks after cancer cell injection, all mice were euthanized, and we collected their plasma and pancreatic, liver, and spleen tissues.

### PDX tumors and tissue microarrays.

We followed our previously published protocol for the heterotopic engraftment of pancreatic patient tumors into immunodeficient mice (63). After harvesting the tissues from the euthanized mice, we embedded them in paraffin and used core samples to construct a tissue microarray.

### Isolation of human neutrophils.

To isolate human neutrophils, we obtained peripheral venous blood samples (10 mL) that were collected from untreated patients with pancreatic cancer and stored at MD Anderson Cancer Center (IRB: PA11-0670). Isolation of the neutrophils was modified as described previously (64). After the plasma and platelets were removed from the blood, the granulocytes were isolated using a Ficoll-Paque PLUS (GE Healthcare, 17-1440-03) with density-gradient centrifugation, and the erythrocytes were removed by 2 rounds of hypotonic lysis with a red blood cell lysis buffer. The viability and purity of the neutrophils were determined by anti–human CD66b PE (clone G10F5; BD Biosciences, 561650) and anti–human CD16 FITC (clone 3G8; BD Biosciences, 560996) double staining using FACS (Supplemental Figure 8).

### In vitro cell invasion assay.

Our cell invasion assay was modified as described previously (21). We suspended 1 × 10^5^ neutrophils in 100 μL of D10 medium and seeded the cells on poly-L-lysine–coated coverslips in a 24-well culture plate for 30 minutes. We then removed the medium and nonadhered neutrophils and added 700 μL of D10 medium with or without IgG, anti-CXCL5 antibody (Abcam, ab9802), BSA, human recombinant CXCL5 (Abcam, ab9803), DNase I (1.5U), apocynin (10 μM; Abcam, ab120615), the PAD4 inhibitor, Cl-amidine (200 μM; Cayman Chemical, 1043444-18-3), and sivelestat (10 μM; ApexBio, B6189). We suspended 2 × 10^5^ cancer cells in 500 μL of serum-free DMEM medium (D0 medium) with or without 7rh, IgG, anti-CXCL5 antibody, BSA, human recombinant CXCL5, DNase I, NADPH oxidase inhibitor, apocynin, PAD4 inhibitor, Cl-amidine, and the neutrophil elastase inhibitor sivelestat and then added the cells to a rehydrated Matrigel chamber (Corning, 354480). After the cells had been cultured for 18 hours at 37°C, the invading cancer cells were fixed with 100% cold methanol and stained with 0.05 mg/mL DAPI (BD Biosciences, 564907). The invading cells in each chamber were counted under a fluorescence microscope, and the average number of cells was calculated based on the number of cells found in 6 fields per chamber.

To generate CCM, we suspended 2 × 10^6^ cancer cells cultured in 2 mL of D0 medium with or without the SYK inhibitor R406 (10 μM; BioVision, 9682); the PKC inhibitor (30 μM; Santa Cruz Biotechnology, sc-3007); another PKC inhibitor, sotrastaurin (2 nM; Selleck Chemicals, S2791); and different dosages of the DDR1 kinase inhibitor, 7rh, for 30 minutes at 37°C. We then seeded the cells on a collagen I-coated 6-well culture plate for 3 hours. Next, we removed and stored the medium at –80°C. NCM was prepared from 1 × 10^6^ neutrophils cultured in 1 mL of D10 medium with or without phorbol 12-myristate 13-acetate (PMA; Sigma-Aldrich, now MilliporeSigma, P8139) for 4 hours; human recombinant CXCL5 for 18 hours; then we collected and stored the medium at –80°C. Neutrophils exposed to NCCM were prepared from 1 × 10^6^ neutrophils cultured in CCM for 18 hours; then we collected the medium with or without heat denaturing (95°C) for 5 minutes, and stored it at –80°C. The NCM or NCCM was added to a 24-well culture plate, and the cancer cells were seeded in D0 medium in a rehydrated Matrigel chamber. After the cells had been cultured for 18 hours at 37°C, the invading cancer cells were fixed with 100% cold methanol and stained with 0.05 mg/mL DAPI. The invading cells in each chamber were counted under a fluorescence microscope, and the average number of cells was calculated based on the number of cells found in 6 fields per chamber.

### Detection of NET structure by immunofluorescence staining.

Neutrophils grown on coverslips were fixed, permeabilized with 4% paraformaldehyde and 0.5% Triton X-100 in PBS, and blocked in PBS containing 2.5% FBS and 2.5% BSA for 1 hour at room temperature. The neutrophils were then incubated overnight at 4°C with primary antibodies: anti–histone H3 (1:50 dilution; Cell Signaling Technology, 3680) and either anti-NE (1:100 dilution; Abcam, ab68672) or anti-myeloperoxidase (1:150 dilution; R&D Systems). After being washed with PBS, secondary antibodies conjugated with Alexa Fluor 488 (Thermo Fisher Scientific, A-11094) or TRITC (Thermo Fisher Scientific) were applied to detect primary Ab (1:250 dilution), and DAPI was used as a counterstain. NET images, defined as areas of colocalized DNA, myeloperoxidase, and citrullinated histone H3, were observed in five fields of each coverslip using a fluorescence microscope, and histone area was used to quantify NET extension using ImageJ software.

For tissue histology, paraffin-embedded sections of tumors were deparaffinized and rehydrated; antigen retrieval was in citrate buffer (10 mM sodium citrate, 0.05% Tween 20, pH 6.0; MilliporeSigma), and the sections were blocked in PBS containing 3% BSA for 1 hour. The tissue sections were then incubated overnight at 4°C with antibodies against myeloperoxidase (1:150 dilution; R&D Systems, AF3667), citrullinated histone H3 antibody (1:100 dilution; Abcam, ab5103), and CK-19 (1:500; Abcam). The tissue sections were then stained with secondary antibodies conjugated with Alex488, TRITC, or Cy5 to detect primary Ab (1:250 dilution), and DAPI was used as a counterstain. NET structure was identified using an automated multispectral imaging microscope (Vectra3, PerkinElmer) and analyzed by using inForm software at the MD Anderson Cancer Center’s Flow Cytometry and Cellular Imaging Core Facility.

### IHC.

Paraffin-embedded sections of tissue obtained from PDX tumors, human tumors in tissue microarrays, or tumors derived from MDA-PATC 148 cells with knockdown DDR1 tumors were deparaffinized and rehydrated; antigen retrieval was in citrate buffer (10 mM sodium citrate, 0.05% Tween 20, pH 6.0). Sections were treated with 3% H_2_O_2_, blocked with Fc Receptor blocker (Innovex) and incubated with 1x blocking buffer (5% BSA in PBS) for 1 hour. The tissue sections were then incubated overnight at 4°C with antibodies against human DDR1 (R&D Systems, AF2396), CXCL5 (Abcam, ab9802), and Ly6G (BD Pharmingen, 551459) antibodies. Biotinylated secondary antibodies (VECTASTAIN ABC kit, Vector Labs) were used for primary antibody detection following the manufacturer’s protocols. Sections were counterstained with hematoxylin. Images were identified using an automated multispectral imaging microscope (Vectra3) and analyzed by using inForm software at the MD Anderson Cancer Center’s Flow Cytometry and Cellular Imaging Core Facility (Supplemental Figure 9).

### shRNAs.

Vectors expressing shRNA against human DDR1 (32: 5′-GACAGCCCATCACCTCTAA-3′ and 33: 5′-CAGGTCCACTGTAACAACA-3′) and mouse DDR1 (588: 5′-TGCAGCTAGAACTTCGCAA-3′ and 809: 5′-AGGTCCTTGGTTACTCTTC-3′) were generated using the pGIPZ-shRNA plasmids (PerkinElmer) and were packaged into lentiviral particles at the MD Anderson Cancer Center’s shRNA and ORFeome Core. Viruses were transfected into BxPC-3 and MDA-PATC 148 cell lines. Puromycin (2 μg/mL; Thermo Fisher Scientific) was used to remove nontransfected cells.

### DDR1 overexpression plasmids.

We packaged pLX304-Blast-V5 vector-expressing human DDR1 ORFs (Dharmacon) into lentiviral particles at MD Anderson Cancer Center’s shRNA and ORFeome Core. Viruses were transfected into Panc1, Miacapa-2, and MDA-PATC 53, 66, 148#32, and 153 cell lines. Blasticidin (0.3 μg/mL; MilliporeSigma) was used to remove nontransfected cells.

### IκB super-repressor mutant plasmids.

IκB super-repressor mutant plasmids were generated using pBABE vectors and were packaged into retrovirus particles at MD Anderson Cancer Center’s shRNA and ORFeome Core. Viruses were transfected into MDA-PATC 148 cells. Puromycin (2 μg/mL) was used to remove nontransfected cells.

### Chemokine and NF-κB phospho antibody arrays.

For the chemokine array, we cultured 1 × 10^6^ cancer cells in 1 mL of D0 medium for 16 hours at 37°C. Then we collected the supernatants and cell lysates so that we could detect the chemokines using a Proteome Profiler Human Chemokine Array Kit (R&D Systems, ARY017).

For the NF-κB phospho antibody array, we suspended 2 × 10^6^ cancer cells in 2 mL of D0 medium and seeded the cells on collagen I-coated 6-well culture plates for 3 hours. The cell lysates were then collected, and we detected the NF-κB pathway using an NF-κB Phospho Antibody Array (Full Moon BioSystems, KAS02).

### ELISA.

We suspended 2 × 10^5^ cancer cells in 200 μL of D0 medium and pretreated the cells with or without SYK inhibitor R406 and the PKC inhibitor sotrastaurin for 30 minutes at 37°C. Then cells were seeded on control or collagen I-coated 96-well plates for 3 hours. Next, the culture supernatants were collected and stored at –80°C. Cells and tumor tissues were lysed and quantified with a Bio-Rad protein assay kit. Culture supernatants, cell lysates, tumor lysates, and plasma were assayed for CXCL5 using a human/mouse CXCL5 immunoassay kit (BioLegend, 440904).

### Real-time PCR.

We quantified mRNA expression using real-time PCR. RNA was prepared using TRIzol Reagent (Invitrogen). We synthesized cDNA using the StepOne Real-Time PCR System (Bio-Rad, 1708840) and analyzed it using StepOne Software v2.2.1 (Bio-Rad). Each sample was tested in triplicate for CXCL1, CXCL2, CXCL3, CXCL5, CXCL6, CXCL7, and CXCL8, and results were normalized by real-time PCR of the cDNA with glyceraldehyde 3-phosphate dehydrogenase (GAPDH). The primers are shown in Supplemental Table 1.

### Detection of neutrophil infiltration in tumors using FACS.

Mice were perfused with a PBS buffer containing heparin (10 U/mL) prior to being euthanized. Then 100 mg of tumor was collected and isolated in single cells by the buffer containing 5 mg/mL collagenase type IV (Gibco, 17104-019) and 1 mg/mL dispase II (Gibco, 17105-041) at 37°C for 2 hours. After 2 hours, tumor homogenates were passed through a 100 μm cell strainer and lysed with a red blood cell lysis buffer to remove the remaining red blood cells. The homogenates were then resuspended in PBS buffer and stained with anti-mouse CD45.1 antibody conjugated with Brilliant Violet 421 (clone A20; BioLegend, 103134), CD11b monoclonal antibody conjugated with APC (M1/70; eBioscience, 17-0112-82), and anti–mouse Ly6G antibody conjugated with APC/Cy7 (clone1A8; BioLegend, 127624). Stained cells were washed twice with FACS buffer and were resuspended in PBS buffer and analyzed. CD45^+^CD11b^+^Ly6G^+^ neutrophil infiltration in tumors was identified using a Beckman Coulter Gallios flow cytometer (Gallios 561) at the MD Anderson Cancer Center’s Flow Cytometry and Cellular Imaging Core Facility. All data were processed using FlowJo software, version 10 (Tree Star Inc.).

### Nuclear protein extraction and immunoblotting.

Cytosolic and nuclear fractions were modified as described previously (65). Cell pellets were lysed in buffer A (20 mM HEPES at pH 7, 10 mM KCl, 2 mM MgCl_2_, 0.5% NP-40, 1 mM NaF, 1 mM Na_3_VO_4_, 1 mM phenylmethylsulfonyl fluoride, and 1 μg/mL aprotinin), homogenized in a glass dounce, and centrifuged at 1500 x *g* for 10 minutes. The supernatant was the cytosolic fraction. The nuclear pellet was isolated and washed 3 times in buffer A. The nuclei were sonicated in RIPA buffer and centrifuged at 15,000 x *g* for 20 minutes. Proteins were quantified with a Bio-Rad protein assay kit. Equal amounts of protein from each sample were subjected to SDS PAGE and transferred to a polyvinylidene membrane (Invitrogen). The membrane was blocked with 5% skim milk in tris-buffered saline with 0.1% Tween 20 for 1 hour. It was then hybridized with DDR1 (D1G6; Cell Signaling, 5583), SYK (Cell Signaling, 2712), phospho-SYK (Cell Signaling, 2710), PKCθ (Cell Signaling, 13643), phospho-PKCθ (Cell Signaling, 9376), NF-κB p65 (Cell Signaling, 6956), phospho-NF-κB p65 (Cell Signaling, 3033), NF-κB p50/100, and β-actin (Sigma-Aldrich, A5316) primary antibodies. The membrane was then incubated with horseradish peroxidase–conjugated secondary antibodies, and the bands were visualized using Western Lightning Plus-ECL (PerkinElmer).

### Chromatin immunoprecipitation assay.

Overall, ChIP assays were modified as described previously (66). An antibody against activated NF-κB p65 antibody (Abcam, ab19870) was used for the ChIP assay. Binding of active p65 to the CXCL5 promoter was quantified by real-time PCR in MDA-PATC 148 cells, with or without DDR1 knockdown. The primers are shown in Supplemental Table 1.

### Statistics.

All data are represented as mean ± SD. For in vitro cell invasion assay, NET extension, and cell stimulation assay, as well as in vivo tissue histology, scores were compared by 1-way ANOVA with Sidak post hoc testing or Student’s *t* test (2 tailed). Fisher’s exact test was used to evaluate the number of mice with liver metastasis. Pearson’s *r* correlation was used to evaluate the association of DDR1/CXCL5, DDR1/Ly6G, and CXCL5/Ly6G histology scores. All the analyses were performed using GraphPad Prism version 8.

### Study approval.

This study was approved by the institutional review board and the institutional animal care and use committee of the University of Texas MD Anderson Cancer Center. Patients gave written informed consent for participation. All animal experiments were conducted according to the National Institutes of Health’s Guide for the Care and Use of Laboratory Animals.

## Author contributions

Conception and design: JBF and JD. Development of methodology: JBF, JD, YK, and CCC. Acquisition of data (provided animals, acquired and managed patients, provided facilities, etc.): JBF, JD, YK, and XL. Analysis and interpretation of data (e.g., statistical analysis, biostatistics, computational analysis): JD, YK, XL, and BD. Writing, review, and/or revision of the manuscript: JBF, JD, HH, and RAB. Administrative, technical, or material support (e.g., reporting or organizing data, constructing databases): JBF, JD, CCC, BD, MHK, TM, MPK, EAK, and RAB. Study supervision: JBF.

## Supplementary Material

Supplemental data

## Version 1 07/08/2021

In-Press Preview

## Version 2 09/08/2021

Electronic publication
