# Supplementary material for: DDR1-induced neutrophil extracellular traps drive pancreatic cancer metastasis
Source: JCI Insight. 2021 Sep 8;6(17):e146133. doi: 10.1172/jci.insight.146133 (PMC8492346; doi:10.1172/jci.insight.146133)
Supplement: Supplemental data [file jciinsight-6-146133-s317.pdf]

Supplementary Figure Legends

Supplementary Figure 1 DDR1 regulated CXCL5 mRNA level in both MDA-PATC 148 and BxPC-3 cells. A DDR1 expression were analyzed by western blotting in MDA-PATC 148 and BxPC-3 cells with shRNA knockdown DDR1 (upper panel) and in MDA-PATC 148$^{KD\#32}$ and BxPC-3$^{KD\#32}$ cells with re-express DDR1 (bottom panel). **B** Neutrophil-related chemokines mRNA level were analyzed by real-time PCR in MDA-PATC 148 and BxPC-3 cells with shRNA knockdown DDR1. Data are mean ± SD. *n* = 3, 3 independent experiments; one-way ANOVA with Sidak post hoc testing, **p*< 0.05, **p<0.01, ***p<0.001. **C** CXCL5 mRNA level by using real-time PCR (left panel) and CXCL5 protein level by using ELISA (right panel) in BxPC-3 cells with knockdown or re-express DDR1 were treated with collagen I for 3 hours. Data are mean ± SD. *n* = 4, 3 independent experiments; one-way ANOVA with Sidak post hoc testing, **p*< 0.05, **p<0.01, ***p<0.001.

Supplementary Figure 2 DDR1 knockdown reduced CD11b+Ly6G+ neutrophils infiltration and liver metastasis. A DDR1 expression were analyzed by western blotting in KP^{wm}C cells with DDR1 knockdown. **B-D** C57BL/6J mice were orthotopically injected with KP^{wm}C (control and 2 of DDR1-deficient clones) cells for 9 weeks. **B** *left:* CD11b+Ly6G+ neutrophils infiltrated into primary tumors were analyzed by FACS. *right:* the calculation of CD11b+Ly6G+ neutrophils infiltration were based on left panel. n=5 mice, data performed in triplicate, one-way ANOVA with Sidak post hoc testing, **p*< 0.05, **p<0.01, ***p<0.001. **C** H&E staining of pancreas and liver section. *Arrow*: region of tumor, Scale bar, 50 µm. The numbers of liver-met, n=5, Fisher's exact test, *p<0.05. **D** ELISA showed CXCL5 level in plasma harvest from mice. n=5 mice, data performed in triplicate, one-way ANOVA with Sidak post hoc testing, **p*< 0.05, **p<0.01, ***p<0.001.

Supplementary Figure 3 DDR1-positive pancreatic cancer cells mediated NET formation from neutrophils and enhanced cancer cell invasion through NADPH oxidase-independently pathway. A-C Human neutrophils were co-culture with DDR1-knockdown of MDA-PATC 148 or BxPC-3 by matrigel transwell chamber, with or without NADPH oxidase inhibitor, PDA4 inhibitor, NE inhibitor and Dase I treatment, for 18 hours. **A** Cit-histone H3 expression were analyzed by western blotting. **B** NET structures were analyzed by immunofluorescence staining using DAPI (*blue*), anti-NE (*red*) and anti-histone (*green*) mAbs. *Scale bar*, 50 µm. **C** The NET quantification is displayed as NET histone area (µm^2) /per 40X filed, 6 fileds per group. Data are mean ± SD. *n* = 5, 3 independent experiments; one-way ANOVA with Sidak post hoc testing, **p*< 0.05, **p<0.01, ***p<0.001.

Supplementary Figure 4 CXCL5 mediated cancer cell invasion through NET. The invaded MDA-PATC 148KD#32 cells were analyzed by matrigel transwell chamber, co-

culture with human neutrophils, with or without recombinant CXCL5, NDAPH oxidase, PDA4, NE inhibitor and DNase I treatment for 18 hours, and the average number of cells was calculated based on the number of cells found in 6 fields per chamber. Data are mean ± SD. $n = 4$, 3 independent experiments; one-way ANOVA with Sidak post hoc testing, *$p < 0.05$, **$p<0.01$, ***$p<0.001$.

Supplementary Figure 5 PKCθ-SYK-NFκB pathway involved in DDR1 induced CXCL5 production and enhanced cancer cell invasion. A Activated NF-kB were analyzed by western blotting using nuclear fraction in MDA-PATC 148 (upper panel) and BxPC-3 cells (bottom panel) with DDR1 knockdown. **B** Top 10 of protein level downregulation by using NFκB phospho antibody array in MDA-PATC 148 cells with DDR1 knockdown. **C** phospho-NF-kB P65 were decected by western blotting in MDA-PATC 148 with IκB super-repressor mutation, with or without TNF-a treatment for 10 mins. **D** CXCL5 protein level were analyzed by ELISA in MDA-PATC 148 with IκB super-repressor mutation, with or without collagen I treatment for 3 hours. **E** CXCL5 protein level were analyzed by ELISA in MDA-PATC 148 (upper panel) and BxPC-3 (bottom panel) with collagen I treatment, with or without R406, PKC inhibitor and Sotrastaurin treatment. **D-E** Data are mean ± SD. $n = 4$, 3 independent experiments; one-way ANOVA with Sidak post hoc testing, *$p < 0.05$, **$p<0.01$, ***$p<0.001$.

Supplementary Figure 6 7rh treatment reduced collagen I-induced CXCL5 production and NET-mediated cell invasion. A CXCL5 protein level were analyzed by ELISA in BxPC-3 with collagen I treatment, with or without 7RH treatment. Data are mean ± SD. $n = 3$, 3 independent experiments; one-way ANOVA with Sidak post hoc testing, *$p < 0.05$, **$p<0.01$, ***$p<0.001$. **B** Human neutrophils were co-culture with MDA-PATC 148 or BxPC-3 by matrigel transwell chamber, with or without 7RH treatment for 18 hours. The number of invaded cells were analyzed by immunofluorescence staining using DAPI and calculated based on the number of cells found in 6 fields /per chamber. Data are mean ± SD. $n = 6$, 3 independent experiments; one-way ANOVA with Sidak post hoc testing, *$p < 0.05$, **$p<0.01$, ***$p<0.001$.

Supplementary Figure 7 Collagen I induced CXCL5 level through STAT3-independent pathway. CXCL5 level were analyzed by ELISA in MDA-PATC 148 and BxPC-3 cells with cucurbitacin I pretreatment for 30 mins, with or without collagen I treatment for 3 hours. Data are mean ± SD. $n = 4$, 3 independent experiments; unpaired two-tailed t-test.

Supplementary Figure 8 Human neutrophils from human blood were checked by FACS using anti-CD16 and anti-CD66b antibodies.

Supplementary Figure 9 Quantification of IHC signal. Images were scanned using PE Vectra3 and processed by inform^R software, the H-Score were calculated by DBA signals/per selected cells. The score of each cells is 0 showed in blue, 1 showed in yellow, 2 showed in orange and 3 showed in brown.

Supplementary Figure 10 Original lumiescence images of the blots presented in the main text. Related to Figure 1-10.

Supplementary Table 1. The primers of real-time PCR and ChiP assay in the main text.

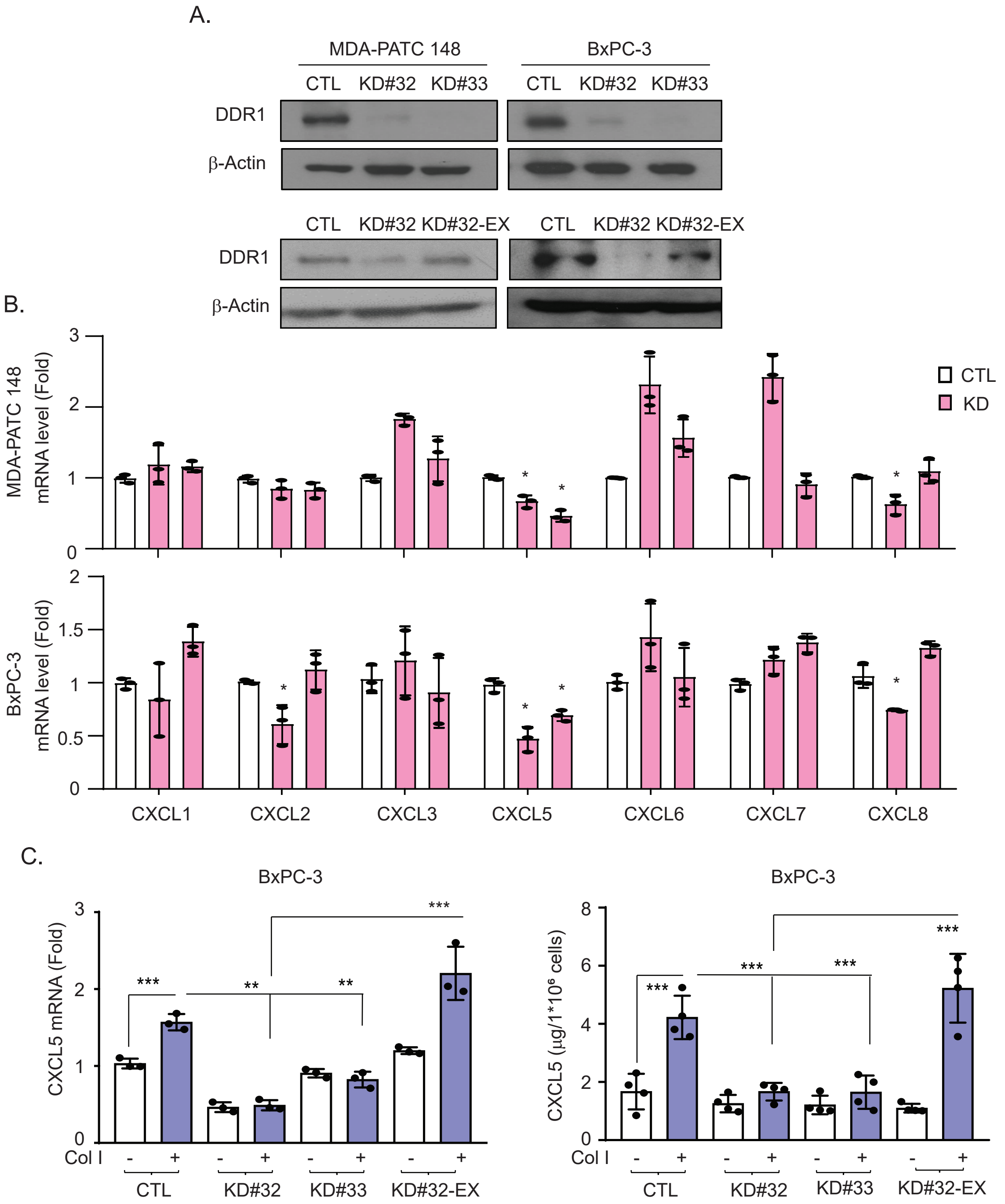

A.
MDA-PATC 148
BxPC-3
CTL KD#32 KD#33
DDR1
β-Actin
CTL KD#32 KD#32-EX
B.
MDA-PATC 148 mRNA level (Fold)
BxPC-3 mRNA level (Fold)
CTL
KD
CXCL1
CXCL2
CXCL3
CXCL5
CXCL6
CXCL7
CXCL8
C.
BxPC-3
CXCL5 mRNA (Fold)
CXCL5 (μg/1*10⁶ cells)
Col I
CTL
KD#32
KD#33
KD#32-EX

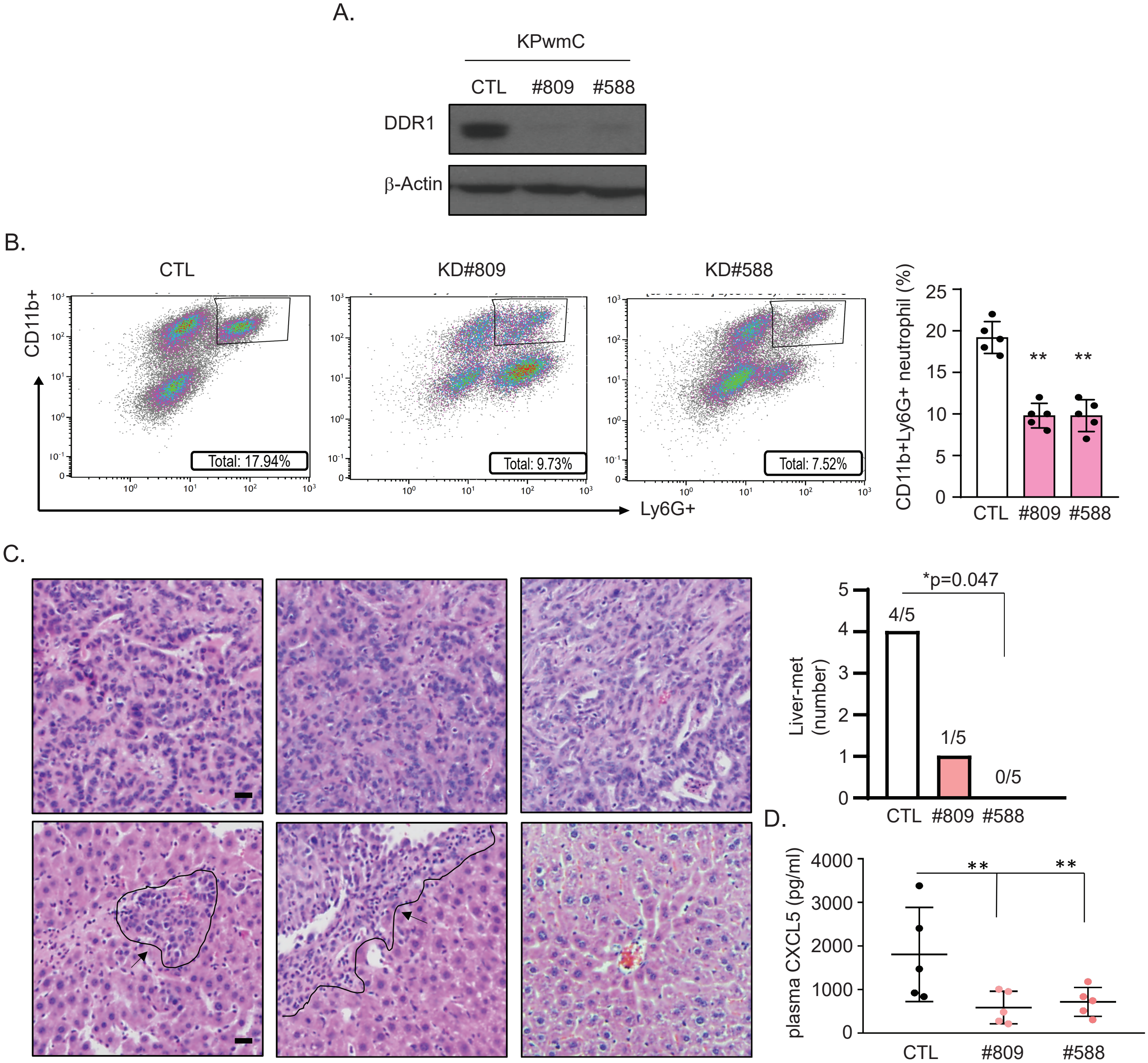
A.
KPwmC
CTL
#809
#588
DDR1
β-Actin
B.
CTL
KD#809
KD#588
CD11b+
Ly6G+
Total: 17.94%
Total: 9.73%
Total: 7.52%
CD11b+Ly6G+ neutrophil (%)
25
20
15
10
5
0
**
**
CTL
#809
#588
C.
*p=0.047
4/5
1/5
0/5
Liver-met (number)
5
4
3
2
1
0
CTL
#809
#588
D.
plasma CXCL5 (pg/ml)
4000
3000
2000
1000
0
**
**
CTL
#809
#588

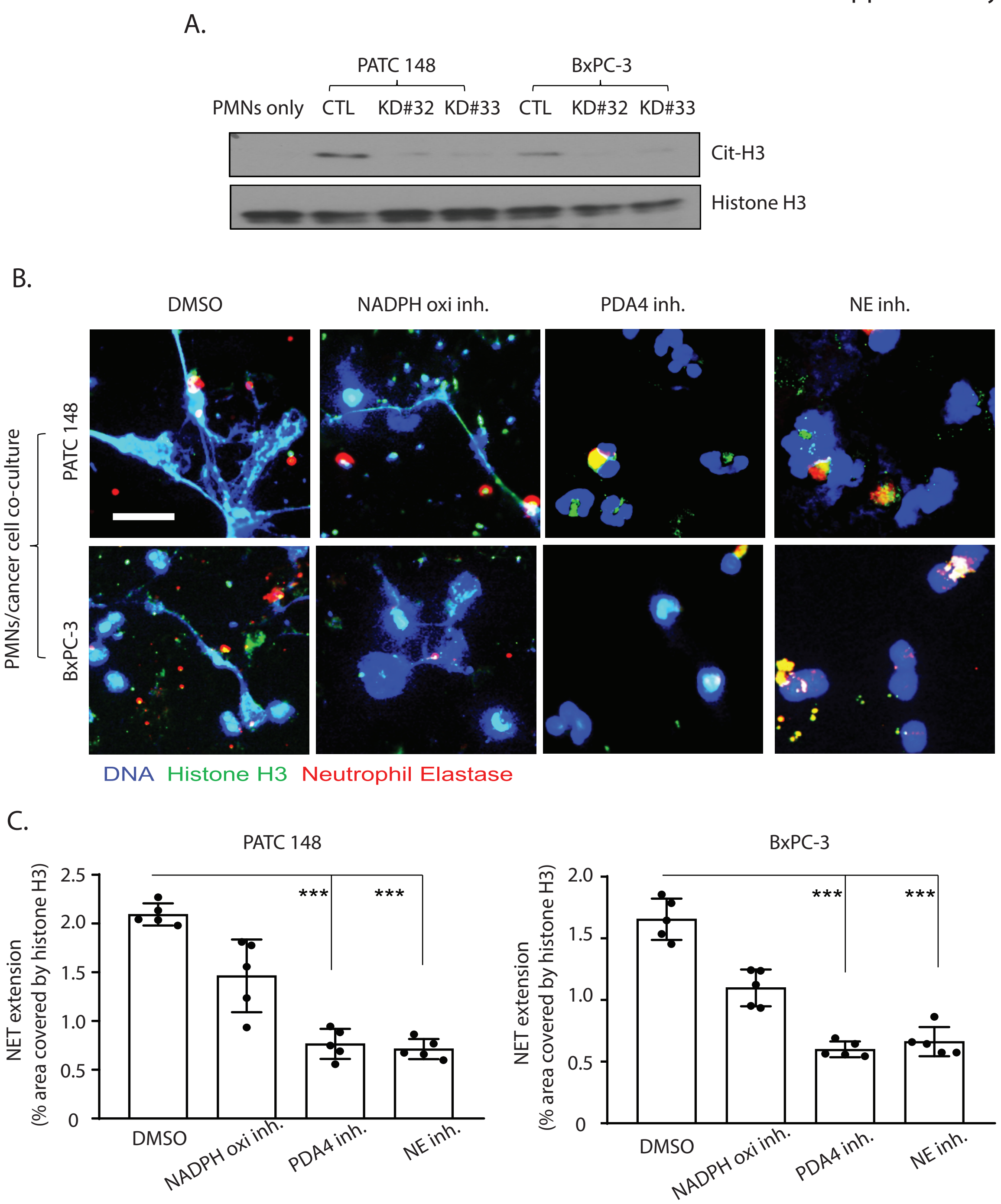
A.
PATC 148
BxPC-3
PMNs only
CTL
KD#32
KD#33
CTL
KD#32
KD#33
Cit-H3
Histone H3
B.
DMSO
NADPH oxi inh.
PDA4 inh.
NE inh.
PMNs/cancer cell co-culture
PATC 148
BxPC-3
DNA
Histone H3
Neutrophil Elastase
C.
PATC 148
BxPC-3
NET extension
(% area covered by histone H3)
2.5
2.0
1.5
1.0
0.5
0
***
***
DMSO
NADPH oxi inh.
PDA4 inh.
NE inh.

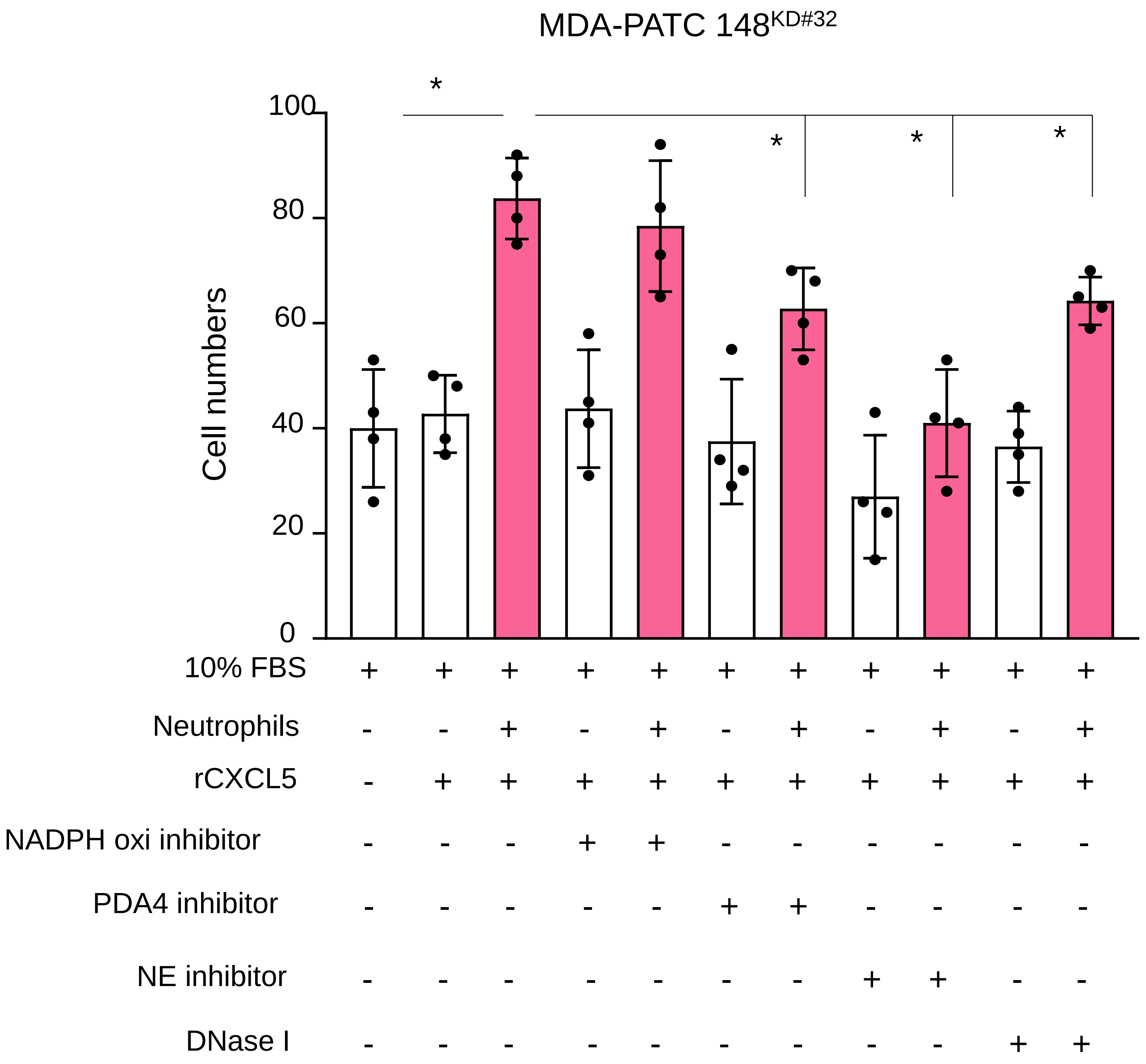
MDA-PATC 148$^{KD\#32}$
Cell numbers
0
20
40
60
80
100
*
*
*
*
10% FBS + + + + + + + + + + +
Neutrophils - - + - + - + - + - +
rCXCL5 - + + + + + + + + + +
NADPH oxi inhibitor - - - + + - - - - - -
PDA4 inhibitor - - - - - + + - - - -
NE inhibitor - - - - - - - + + - -
DNase I - - - - - - - - - + +

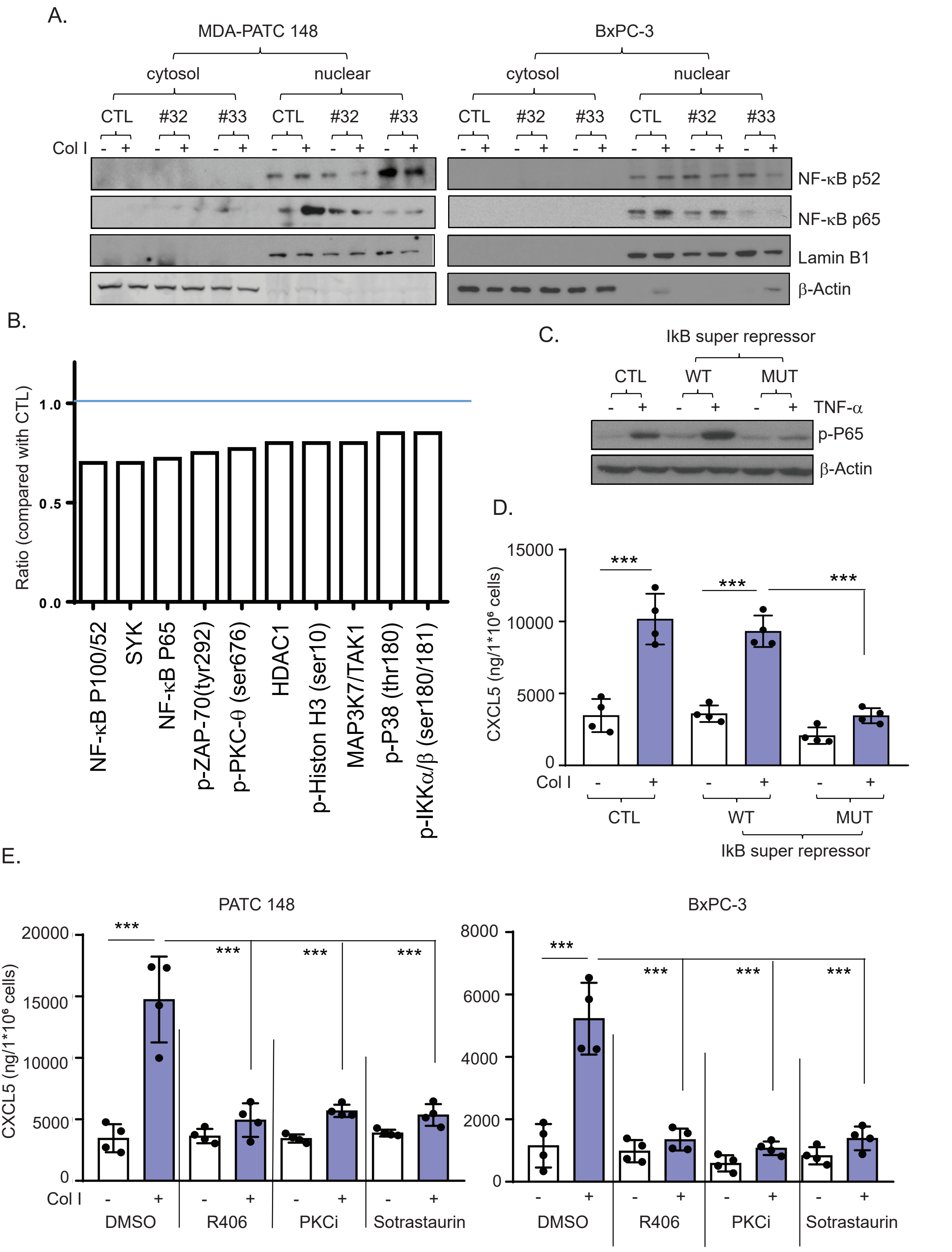
A.
MDA-PATC 148
BxPC-3
cytosol
nuclear
CTL
#32
#33
Col I
NF-κB p52
NF-κB p65
Lamin B1
β-Actin
B.
Ratio (compared with CTL)
1.0
0.5
0.0
NF-κB P100/52
SYK
NF-κB P65
p-ZAP-70(tyr292)
p-PKC-θ (ser676)
HDAC1
p-Histon H3 (ser10)
MAP3K7/TAK1
p-P38 (thr180)
p-IKKα/β (ser180/181)
C.
IkB super repressor
CTL
WT
MUT
TNF-α
p-P65
β-Actin
D.
CXCL5 (ng/1*10⁶ cells)
15000
10000
5000
0
***
Col I
CTL
WT
MUT
IkB super repressor
E.
PATC 148
BxPC-3
CXCL5 (ng/1*10⁶ cells)
20000
15000
10000
5000
0
8000
6000
4000
2000
***
Col I
DMSO
R406
PKCi
Sotrastaurin

A.

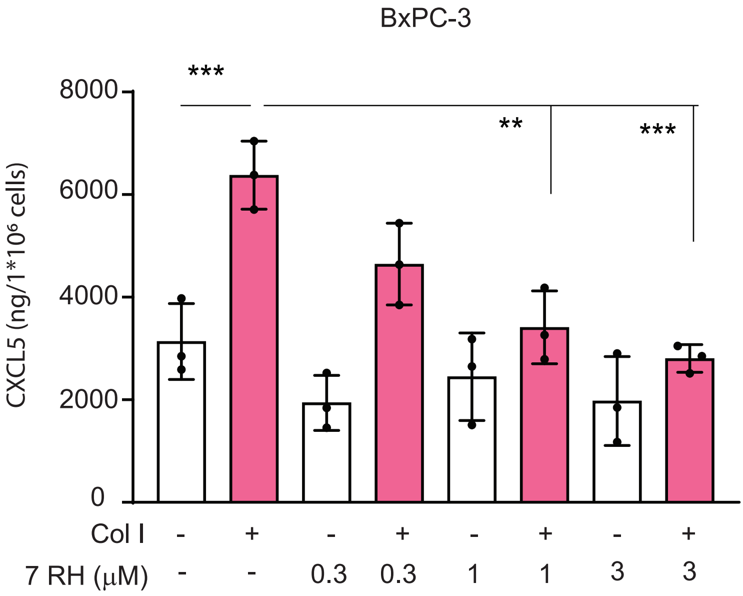
BxPC-3
CXCL5 (ng/1*10⁶ cells)
8000
6000
4000
2000
0
***
**
***
Col I - + - + - + - +
7 RH (μM) - - 0.3 0.3 1 1 3 3

B.

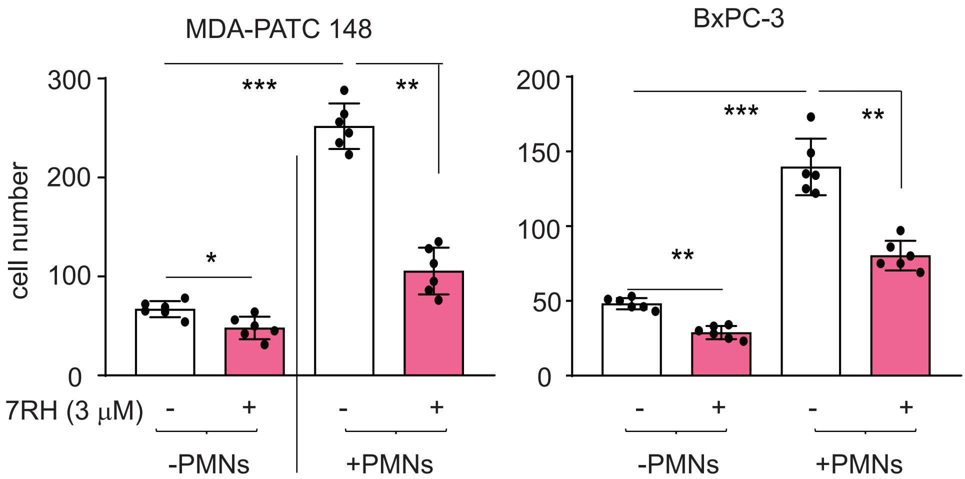
MDA-PATC 148
cell number
300
200
100
0
***
**
*
7RH (3 μM) - + - +
-PMNs
+PMNs
BxPC-3
200
150
100
50
0
***
**
**
- + - +
-PMNs
+PMNs

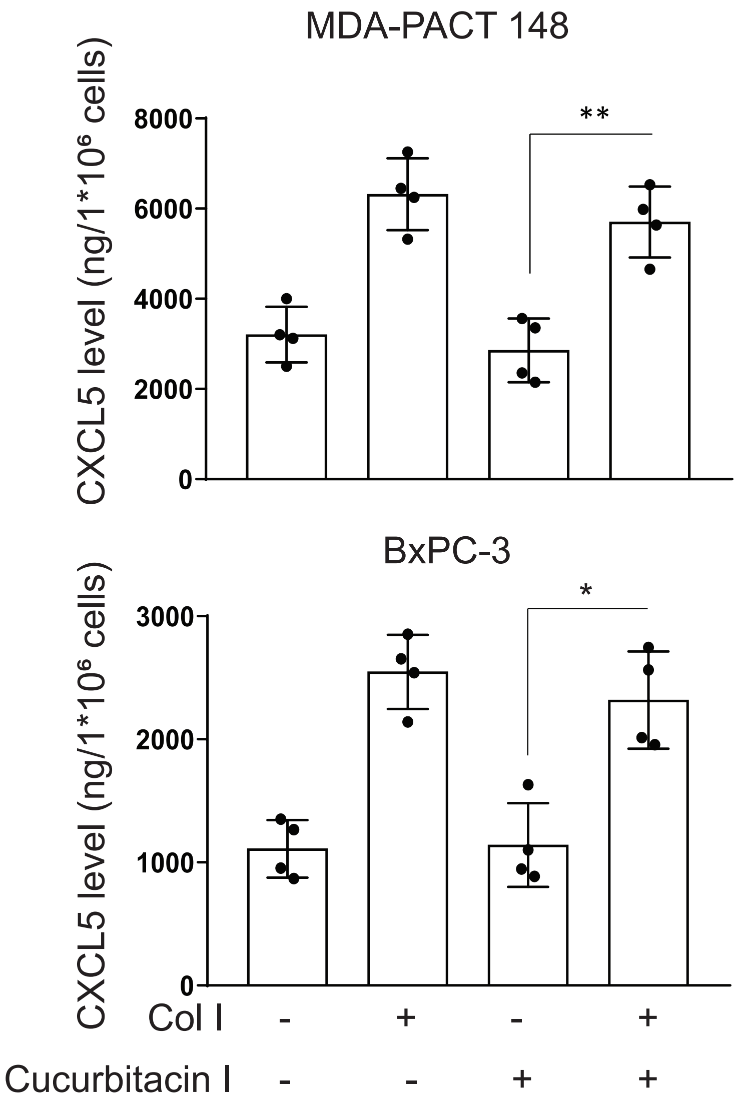
MDA-PACT 148
CXCL5 level (ng/1*10⁶ cells)
8000
6000
4000
2000
0
**
BxPC-3
CXCL5 level (ng/1*10⁶ cells)
3000
2000
1000
0
*
Col I - + - +
Cucurbitacin I - - + +

Supplementary Figure 8

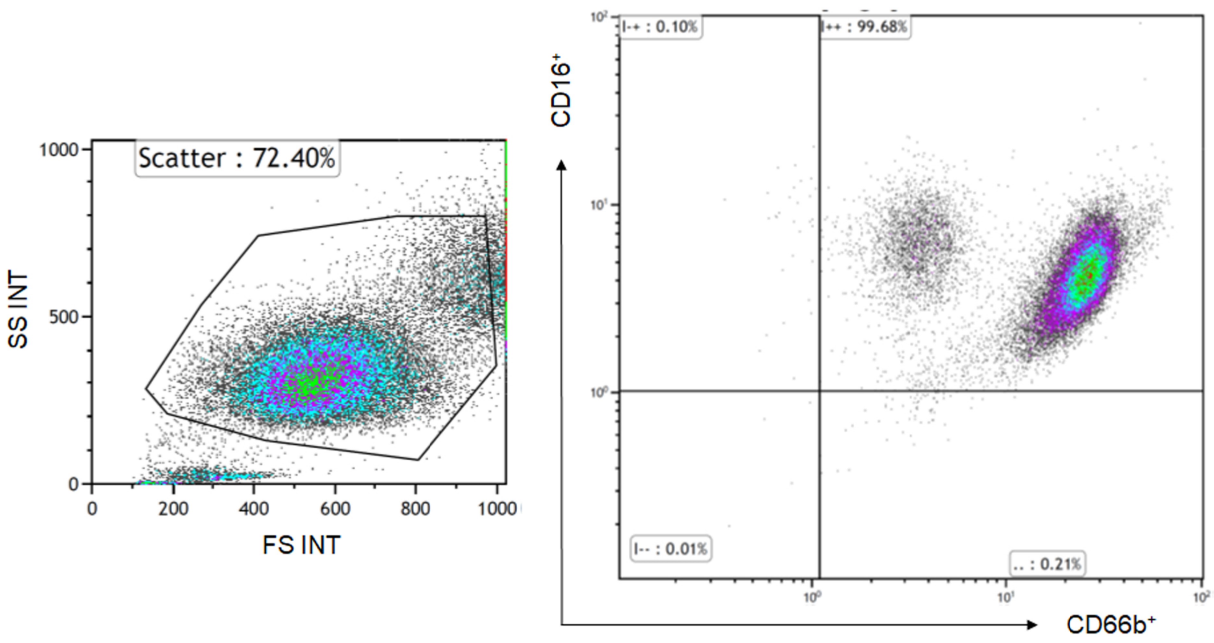

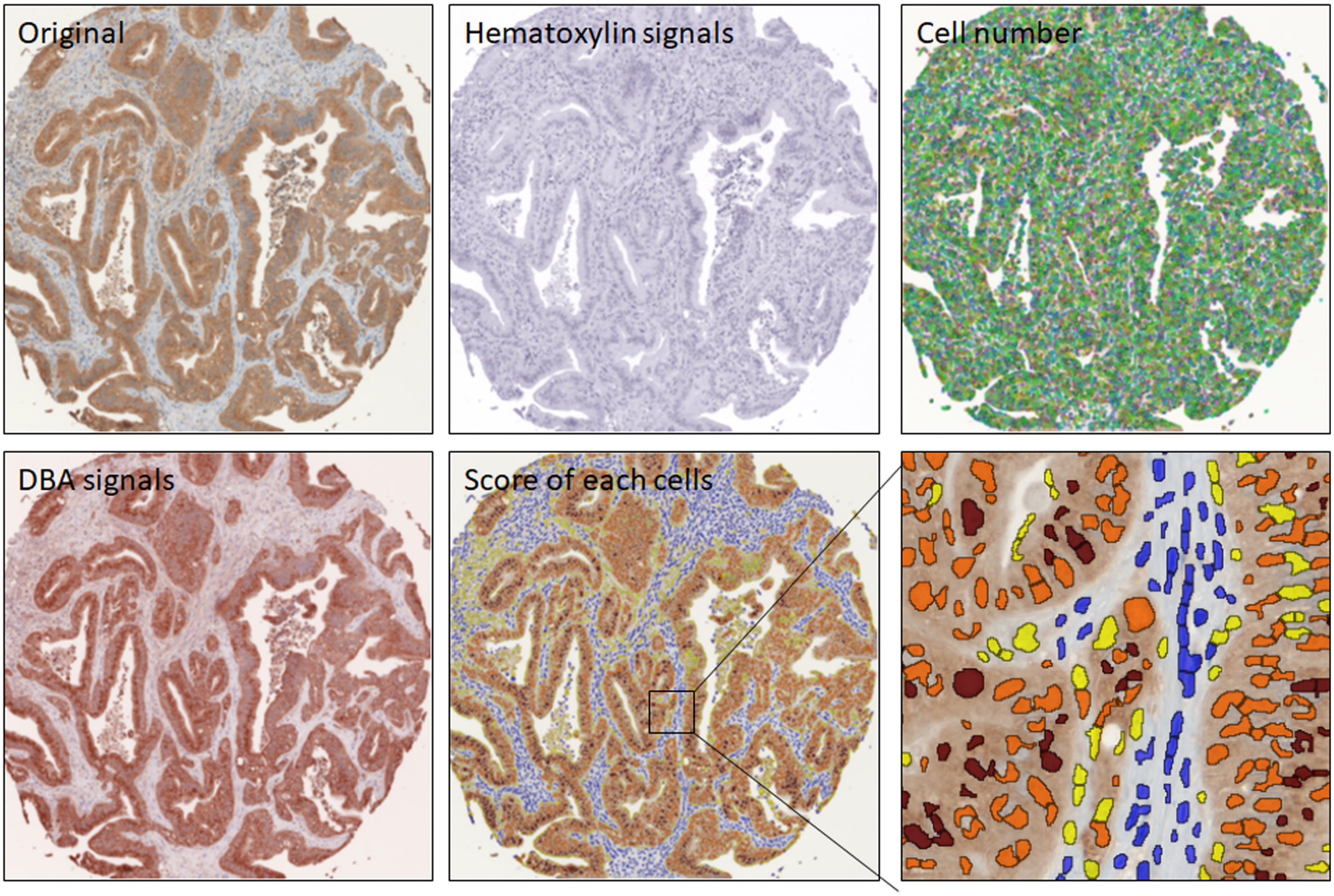
Original
Hematoxylin signals
Cell number
DBA signals
Score of each cells

Supplementary Figure 10

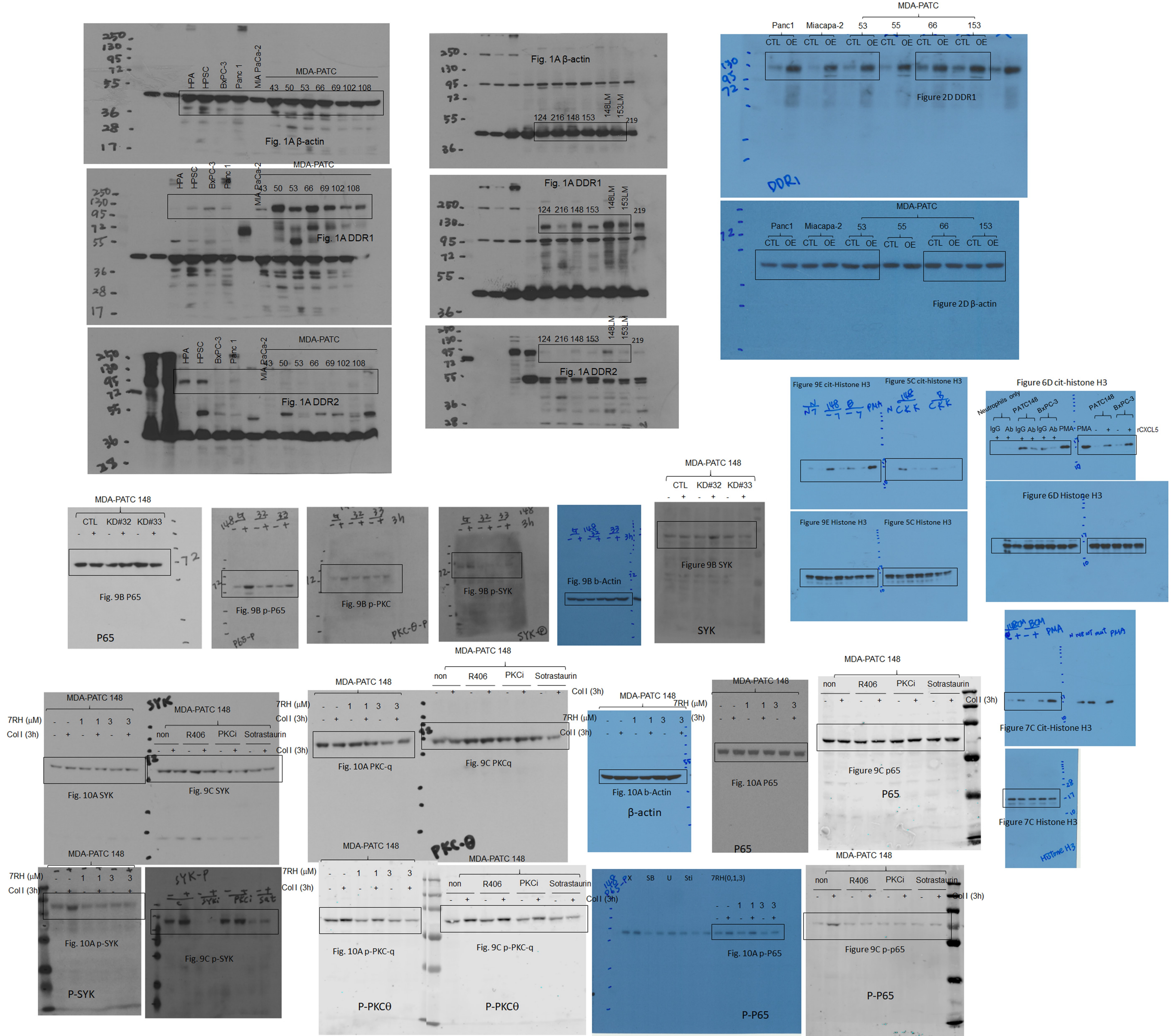

Supplementary Table 1. The primers of real-time PCR and ChiP assay in the main text.

Primer for real-time PCR	Sequence 5' to 3'
hCXCL1-F	GAAAGCTTGCCTCAATCCTG
hCXCL1-R	CTTCCTCCTCCCTTCTGGTC
hCXCL2-F	GGGCAGAAAGCTTGTCTCAA
hCXCL2-R	GCTTCCTCCTTCCTTCTGGT
hCXCL3-F	CGCCCAAACCGAAGTCATAG
hCXCL3-R	GCTCCCCTTGTTCAGTATCTTTT
hCXCL5-F	TGGACGGTGGAAACAAGG
hCXCL5-R	CTTCCCTGGGTTCAGAGAC
hCXCL6-F	AGAGCTGCGTTGCACTTGTT
hCXCL6-R	GCAGTTTACCAATCGTTTTGGGG
hCXCL7-F	GGCTTCCTCCACCAAAGGAC
hCXCL7-R	TCTTTGCCTTTCGCCAAGTT
hCXCL8-F	GAATGGGTTTGCTAGAATGTGATA
hCXCL8-R	CAGACTAGGGTTGCCAGATTTAAC
hGAPDH-F	ACGGATTTGGTCGTATTGGG
h-GAPDH-R	TGATTTTGGAGGGATCTCGC
Primer for ChIP assay	
CXCL5 promoter-NFkB-f-1	TAGAGGTGCACGCAGCTCCT
CXCL5 promoter-NFkB-r-1	GAGCACTGTGGCTTCCTCGT
